# RNA structure profiling at single-cell resolution reveals new determinants of cell identity

**DOI:** 10.1038/s41592-023-02128-y

**Published:** 2024-01-04

**Authors:** Jiaxu Wang, Yu Zhang, Tong Zhang, Wen Ting Tan, Finnlay Lambert, Jefferson Darmawan, Roland Huber, Yue Wan

**Affiliations:** 1 Stem Cell and Regenerative Biology, Genome Institute of Singapore, A*STAR, Singapore, Singapore; 2 Division of Biomedical Sciences, Warwick Medical School, University of Warwick, Coventry, UK; 3 Bioinformatics Institute, A*STAR, Singapore, Singapore; 4 Department of Biochemistry, National University of Singapore, Singapore, Singapore

**Keywords:** RNA sequencing, RNA, Differentiation

## Abstract

RNA structure is critical for multiple steps in gene regulation. However, how the structures of transcripts differ both within and between individual cells is unknown. Here we develop a SHAPE-inspired method called single-cell structure probing of RNA transcripts that enables simultaneous determination of transcript secondary structure and abundance at single-cell resolution. We apply single-cell structure probing of RNA transcripts to human embryonic stem cells and differentiating neurons. Remarkably, RNA structure is more homogeneous in human embryonic stem cells compared with neurons, with the greatest homogeneity found in coding regions. More extensive heterogeneity is found within 3′ untranslated regions and is determined by specific RNA-binding proteins. Overall RNA structure profiles better discriminate cell type identity and differentiation stage than gene expression profiles alone. We further discover a cell-type variable region of 18S ribosomal RNA that is associated with cell cycle and translation control. Our method opens the door to the systematic characterization of RNA structure–function relationships at single-cell resolution.

## Abstract (web summary)

Single-cell structure probing of RNA transcripts enables simultaneous determination of transcript secondary structure and abundance in single cells, allowing new insights into RNA structural heterogeneity within and among cells.

## Main

Understanding the genetic and transcriptomic determinants of cell identity remains a key question in biology. Toward this goal, numerous groups have developed technologies to interrogate different aspects of the genome and transcriptome in a single cell, including chromatin states, RNA abundance levels and alternative splicing^1–5^. RNA structure plays critical roles in every step of an RNA’s lifecycle, including transcription, splicing, localization^6,7^, translation^8^ and RNA decay^9^. However, how RNA structure differs among—and contributes to—the identity of individual cells is poorly understood. As such, defining the structurome of an individual cell will greatly enhance our understanding of cellular identity and is a fundamental problem that needs to be addressed.

The brain is one of the most complex organs in our body and undergoes extensive co-/posttranscriptional gene regulation^10–12^. Recent advances in single-cell transcriptomic analyses have revealed a large amount of diversity in posttranscriptional processes, including RNA expression, alternative splicing and alternative 3′ untranslated region (UTR) usage in individual cells^13^. However, the extent to which RNA structures differ, are regulated and have different functions in individual cells remains unknown. As such, being able to probe RNA structures in single cells during the neuronal differentiation process deepens our understanding of neuronal states during brain development.

Unfortunately, current single-cell RNA sequencing cannot be directly applied to study RNA structure. Recent developments in high-throughput RNA structure mapping technologies such as DMS-mutational mapping, icSHAPE and SHAPE-MaP have enabled us to probe the structures of thousands of RNAs simultaneously, providing insights into the role of RNA structures in diverse organisms and cellular states^14–19^. However, the requirement for large amounts of starting material (typically from 10^7^ cells) makes it difficult to assess the diversity of RNA structures in small cellular populations or single cells, limiting our understanding of RNA structure and function.

**Fig. 1:**
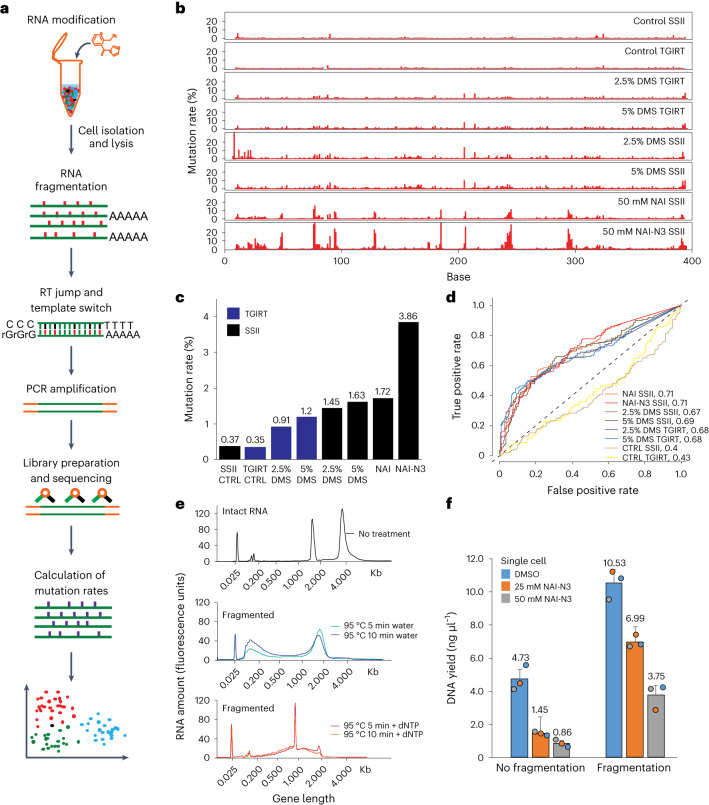
Development of sc-SPORT to probe RNA structures in single cells. **a**, Experimental workflow of library preparation of sc-SPORT. **b**, Bar plots showing detected mutation rates on the Tetrahymena ribozyme using different RT enzymes (SSII and TGIRT) and different RNA modification compounds (DMS, NAI and NAI-N3) at different concentrations. **c**, A bar plot showing the average mutation rates along single-stranded regions of Tetrahymena ribozyme upon different treatments in **b**. **d**, ROC curves showing the accuracy of different treatments against the known secondary structure of Tetrahymena ribozyme. **e**, Bioanalyzer plots showing the size distribution of untreated RNA (top), RNA fragmented in water (middle) and RNA fragmented in water in the presence of 1 mM dNTP (bottom). **f**, Bar plots showing the amount of DNA that is generated from PCR amplification in single cells before (left) and after fragmentation (right) and in the presence of different concentrations of NAI-N3. The center represents the mean, and the error bars show the standard deviation. *N* = 3 biological replicates. Source data

In this Article, we developed a new method—single-cell structure probing of RNA transcripts (sc-SPORT)—to simultaneously determine RNA secondary structure and gene expression information in a single cell (Fig. 1a). We show that sc-SPORT is accurate and applied it to study RNA structures in human embryonic stem cells (hESCs) and different stages of neuronal differentiation. We observed that individual transcripts can take on a variety of structures in different cells, differences in structural similarity can be regulated by RNA-binding proteins and structural variation in single cells can better define cellular identities. These single-cell studies provide first glimpses into the nature of RNA structure dynamics, regulation and function inside individual cells during neurogenesis.

## Results

### Sc-SPORT detects RNA structures in single cells

To perform single-cell RNA structure probing, we modified RNA in single-stranded regions using structure-probing compounds. We then isolated individual cells, lysed and fragmented the RNAs, enriched for poly(A)^+^ RNAs, and performed reverse transcription (RT) and PCR amplification (Fig. 1a). After high-throughput sequencing and mapping to the transcriptome, we calculated the mutation rate at each base. A high mutation rate represents a high accessibility of the structure probing compound and indicates increased single strandedness, while a low mutational rate represents a low accessibility of the structure-probing compound for the base and indicates decreased accessibility at the base (Methods).

**Extended Data Fig. 1:**
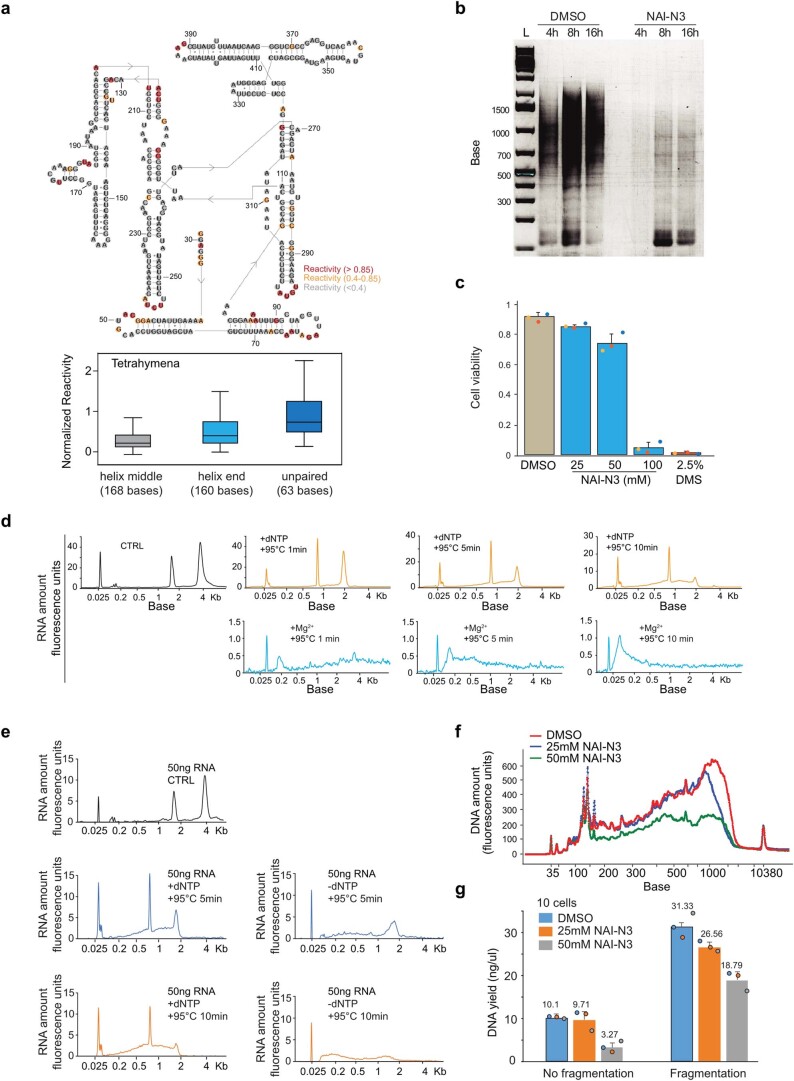
Optimization of the experimental sc-SPORT workflow. **a**, Top: NAI-N3 reactivity of the Tetrahymena ribozyme was mapped to its secondary structure. The reactivity was normalized as previously described. Bottom: Boxplot showing the distribution of reactivities for bases in base-paired between adjacent base pairs (left), paired in terminal base pair (middle), and unpaired regions (right) along the Tetrahymena ribozyme RNA. The nucleotide numbers are as shown. The box plots show the mean and 25^th^ to 75^th^ percentile inter-quartile range, the bars show the range from 5^th^ to 95^th^ percentile. **b**, Gel image showing the distribution of the length of cDNA fragments after 4, 8, and 16 hours of reverse transcription in DMSO and NAI-N3 treated RNAs. The first lane shows the DNA ladder (bases). **c**, Barplot showing the average fraction of viable cells after treatment with DMSO (grey), NAI-N3 (blue), and DMS (orange) at various concentrations. The center represents mean, and the error bars show the standard deviation. N = 4 biological replicates. **d**, Line plots showing the distribution of 5 ng hESC total RNA segment lengths before (black) and after fragmentation, by dNTP (orange) and Mg2+ (blue), under different conditions. **e**, Bioanalyzer plots showing the size distribution of 50 ng hESC total RNA segment lengths before (top) and after fragmentation with 1 mM dNTP (Middle left and Bottom left), or without dNTP (Middle right and Bottom right). **f**, Density plot of the RNA fragment length distribution treated with either DMSO (red), 25 mM NAI-N3 (blue), or 50 mM NAI-N3 (green) using Agilent Bioanalyzer. **g**, Barplot showing the amount of DNA generated after library preparation when the starting RNA is unfragmented (left) or fragmented (right). Starting RNA is from 10 cells and is either treated with DMSO (blue), 25 mM NAI-N3 (orange), or 50 mM NAI-N3 (grey). The center represents the mean, and the error bars show the standard deviation. N = 3 biological replicates. Source data

Low modification and mutation rates along an RNA make it difficult to assess chemical-induced mutations in single-cell RNA structure probing. To identify conditions that result in high mutation rates, we tested several in vivo structure probing compounds together with different RT enzymes along the Tetrahymena ribozyme, which has a well-known secondary structure in vitro. We observed that the treatment of RNAs with NAI-N3, a cell-permeable SHAPE compound^16^, together with RT using Superscript II (SSII), resulted in high mutation rates (3.86%; Fig. 1b,c) and is highly accurate in structure determination (Fig. 1d and Extended Data Fig. 1a). As SSII tends to result in very slow rates of RT in the presence of manganese, we used a long RT time to enable efficient complementary DNA production and PCR amplification (Extended Data Fig. 1b). We also confirmed that the NAI-N3 concentration used in vivo does not result in cell death of hESCs (Methods and Extended Data Fig. 1c).

Another major challenge in developing single-cell RNA structure probing is that chemical modifications introduced on an RNA result in an increase in premature reverse transcriptase drop-offs. As such it is difficult for the RT enzyme to travel to the beginning of the transcript to undergo template switching for second-strand synthesis, resulting in low library yield. As most of the current fragmentation methods are designed for larger amounts of RNAs (>100 ng), it is challenging to fragment small amounts of RNAs (10 pg to 1 ng) to sizes of interest. To increase the efficiency of second-strand synthesis and library preparation, we tested different RNA fragmentation conditions to gently reduce the RNA length of long RNAs without breaking the shorter RNAs (Extended Data Fig. 1d). Surprisingly, we observed that the fragmentation at 95 °C in the presence of deoxyribonucleotides (dNTPs) for small RNA amounts resulted in the size distribution of RNA fragments that are centered around 1,000 bases (Fig. 1e and Extended Data Fig. 1d–f). The presence of dNTPs also enabled us to continue with RT directly, without introducing an additional purification step, saving time and material. This mild fragmentation step enabled an increase in the amount of cDNA product generated from ten cells and single cells (Fig. 1f and Extended Data Fig. 1g), allowing us to now perform single-cell RNA structure probing.

**Fig. 2:**
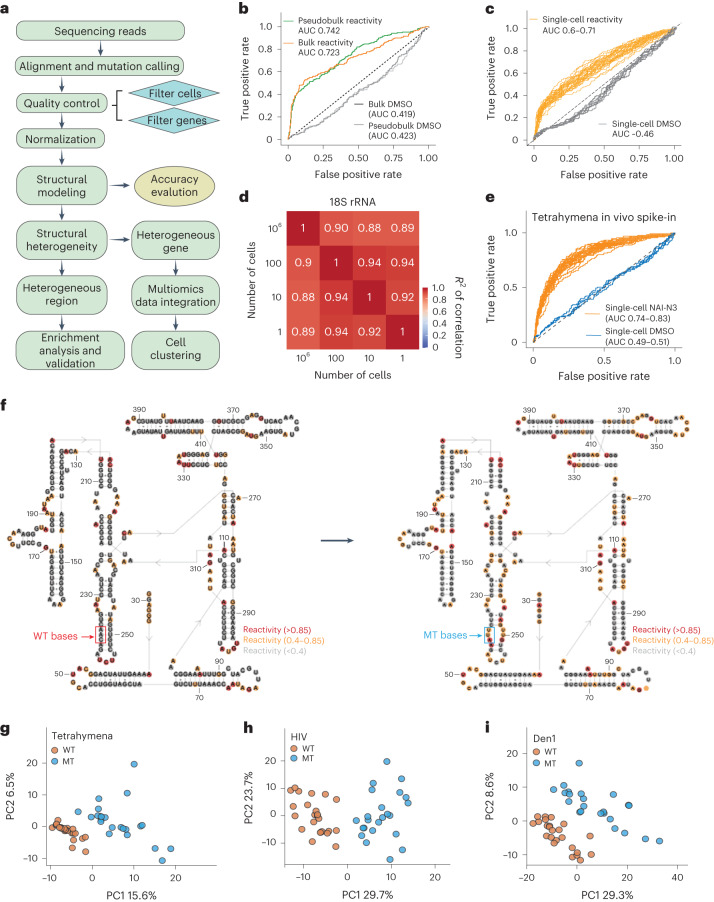
Sc-SPORT can measure single-cell RNA structure accurately. **a**, The bioinformatic workflow for analyzing sc-SPORT data. **b**, ROC curves showing the accuracy of bulk and pseudobulk reactivity for 18S rRNA obtained from single cells. **c**, ROC curves showing the accuracy of single-cell reactivity for 18S rRNA in hESCs. The reactivity is calculated from 40 NAI-N3-treated cells and 7 DMSO-treated cells in total. **d**, A heatmap showing the Pearson correlation of structure reactivities for 18S rRNA between millions (10^6^), 100, tens (10) and the pseudobulk of 40 single cells (1), The sample number for each condition is as shown. **e**, ROC curves show the accuracy of reactivity at single-nucleotide resolution for the Tetrahymena ribozyme, which was transfected into single cells. The reactivities come from 24 NAI-N3-treated cells and 4 DMSO-treated cells. **f**, Pseudobulk reactivities of the transfected WT (left) and MT (right, bases 237–239) Tetrahymena ribozyme in HEK293T cells mapped onto the secondary structure of the Tetrahymena ribozyme. We performed gene-level normalization for the Tetrahymena ribozyme in each cell. **g**–**i**, Principal component analysis of single cells based on the structure reactivity of the transfected RNAs. Scatter plots of reactivity in individual cells based on whether they contain the WT (orange) or MT (blue) structures of Tetrahymena ribozyme (**g**), HIV RNA (**h**) or Den1 (**i**). There are 48 cells in total, half of them were transfected with WT and the other half with MT RNAs.

**Extended Data Fig. 2:**
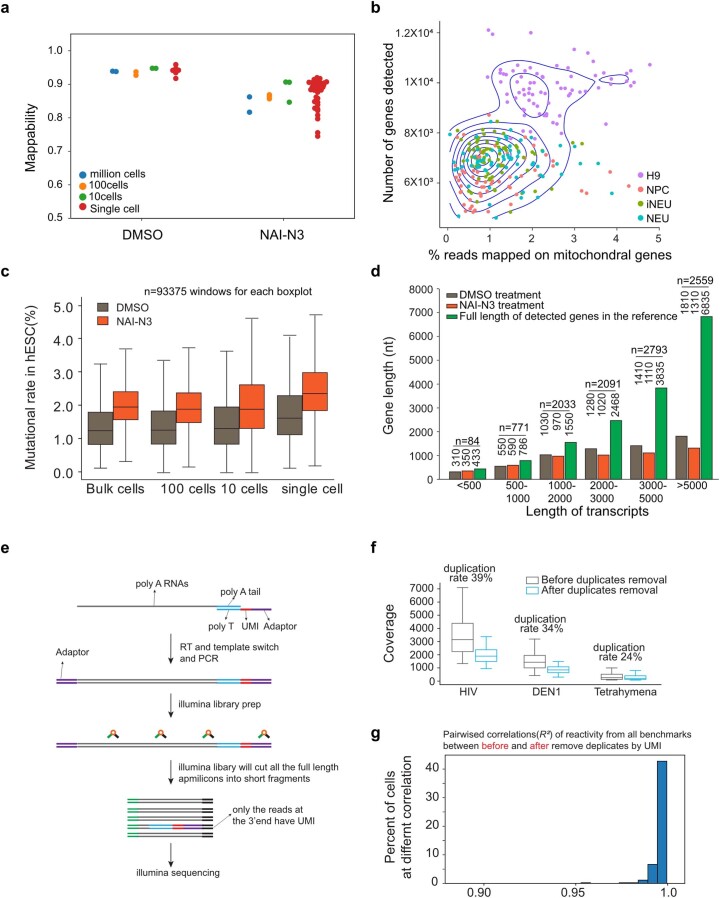
Determining the data quality of sc-SPORT. **a**, Distribution of the fraction of mapped sequencing reads from hESC libraries constructed from millions of cells, 100 cells, 10 cells, and single-cell. DMSO and NAI-N3 treated cells are on the left and right, respectively. **b**, A joint scatter plot showing the percentage of reads mapped to mitochondrial genes (X-axis) and the number of transcripts detected (Y-axis) in each cell. Different time points are labeled with different colors. **c**, The mutant rates of each 10-nt window present in hESC transcripts when treated with NAI-N3 or DMSO, in bulk, 100-cells, 10-cells, and single-cell samples respectively. The boxplots show the means and 25th to 75th percentile inter-quartile range, the bars show the range from 5^th^ to 95^th^ percentile. The number of 10-nt windows is N = 93375 for each boxplot. **d**, Barplot showing the average detected gene lengths grouped by the gene lengths in DMSO and NAI-N3 treated samples. The annotated transcript length is green, the detected length from DMSO-treated libraries is in brown, and the detected length from NAI-N3-treated libraries is in orange. The genes were assigned to their groups according to the size of their full-length transcript. The detected regions were defined as regions with more than 600 reads in at least one cell in 76 hESC cells. The number of transcripts for each group is labeled on top of the bars. **e**, Schematic showing the single-cell RNA structure workflow and the locations of the UMIs when they are incorporated into the protocol. **f**, Boxplots showing the distribution of read depth on the 3’end of HIV, DEN1, and Tetrahymena ribozyme before (black) and after (blue) duplicate removal. The duplication rates of these three transfected RNAs range from 24–39%. The boxplots show the means and 25th to 75th percentile inter-quartile range, the bars show the range from 5^th^ to 95^th^ percentile. The boxplot represents read coverage from N = 24 single cells for each gene. **g**, Histogram showing the distribution of SHAPE-reactivity correlation before and after duplicate removal.

To quantitate RNA structure information in single cells, we developed a computational pipeline to analyze sc-SPORT data (Fig. 2a). We observed high levels of mappability of sequencing reads from samples that are generated from millions of cells to single cells (median mappability >0.85; Extended Data Fig. 2a and Supplementary Table 1), suggesting that our single-cell structure libraries are of good quality. As cells with poor sequencing quality typically contain high mitochondrial RNA amounts and few detected genes, we filtered out cells with fewer than 5,000 detected genes and more than 5% mitochondrial RNA (Extended Data Fig. 2b). We then calculated the RNA structural reactivities in each cell (Methods) and observed a 2–3% modification rate in both bulk and single cells with NAI-N3 treatment (Extended Data Fig. 2c). Finally, we obtained sequencing reads of around 1 kb from the 3′ end of the transcript (Extended Data Fig. 2d), as expected from the RNA size distribution after fragmentation (Extended Data Fig. 1f).

While, traditionally, unique molecular identifiers (UMIs) are added to single-cell RNA sequencing libraries to collapse PCR duplicates, our current library preparation strategy will only contain UMIs at the end of the transcript due to a tagmentation step before final library amplification (Extended Data Fig. 2e). As such, we did not include UMIs for our single-cell RNA structure libraries. However, to test the duplication rates of our library, we added UMIs at the ends of the transcripts for 48 single cells. After PCR duplicate removal, we observed duplication rates of 24–39% for three transfected RNAs in our sc-SPORT library (Extended Data Fig. 2f). This falls within the usual range of duplication rates for single-cell RNA sequencing libraries, indicating that our libraries are not suffering from severe bottlenecking effects. Importantly, using the UMIs, we observed that reads with or without duplicate removal show a high correlation in their modification rates (Extended Data Fig. 2g), suggesting that duplication removal does not affect our SHAPE-reactivity calculation.

**Extended Data Fig. 3:**
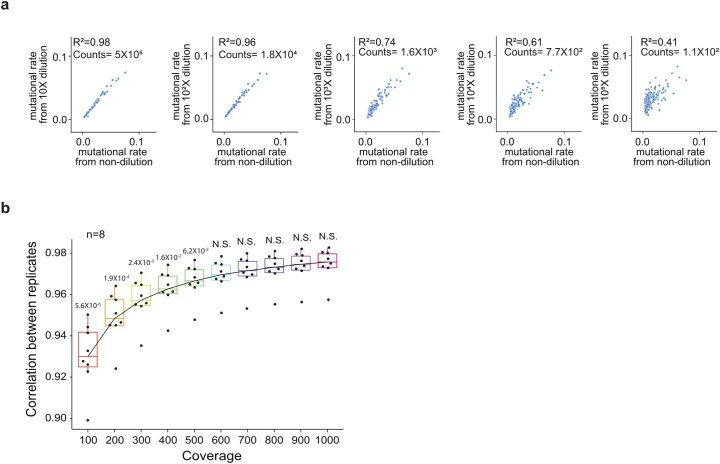
Determining minimal read depth for sc-SPORT downstream analysis. **a**, *In vitro* serial dilution and structure mapping of the Tetrahymena ribozyme. Scatterplots show per base SHAPE-reactivity correlation between 5×10^5, 1.8×10^4, 1.6×10^3, 7.7×10^2 and 1.1×10^2 to 10^7 copies of Tetrahymena ribozyme along the RNA. **b**, Boxplot showing the distribution of correlation of mutation rates between two technical replicates at different coverage depths for a window size of 10 nucleotides. The correlation at a coverage filter of 600X becomes similar to that of a coverage filter of 1000X, and we used this filter for all our downstream analyses. *P*-values were calculated using a two-sided Student’s T-test. The boxplots show the means and 25th to 75th percentile inter-quartile range, the bars show the range from 5^th^ to 95^th^ percentile. N = 8 biological replicates.

To determine the depth of sequencing required to calculate accurate single-cell RNA structure data, we performed in vitro structure mapping on a series of tenfold dilutions of the Tetrahymena ribozyme and observed good structural correlation with bulk at ∼700 copies of RNAs in solution (Extended Data Fig. 3a). Additionally, we subsampled our single-cell RNA structure probing data and compared the reactivity signals obtained at different sequencing depths between technical replicates of sc-SPORT. We observed a good correlation at a depth of 600 reads per 10 bases (Extended Data Fig. 3b), confirming that this is a good cutoff for our downstream analysis in studying single-cell structures.

**Extended Data Fig. 4:**
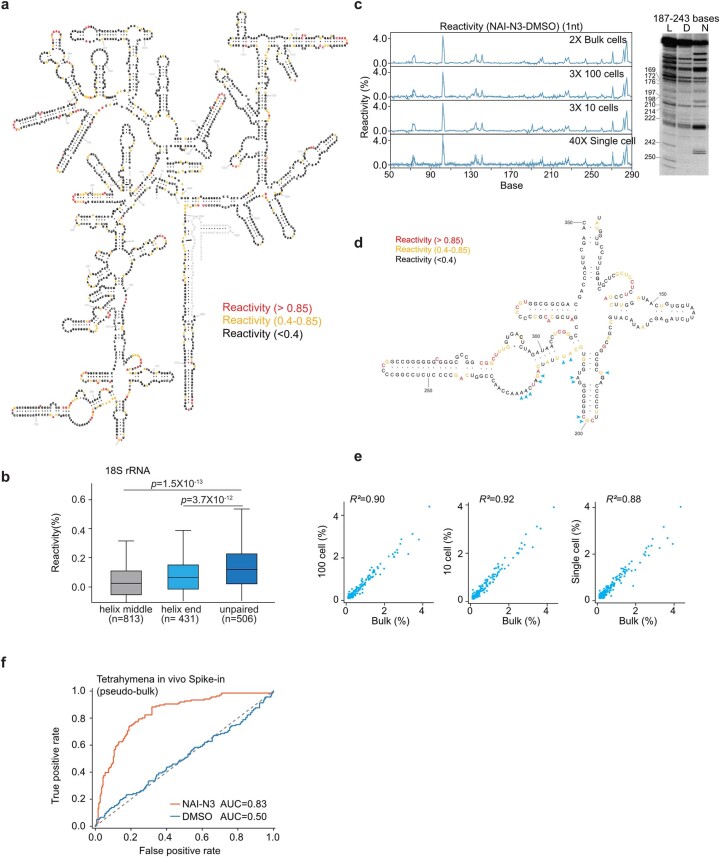
sc-SPORT can identify RNA structures of 18S rRNA. **a**, Pseudobulk reactivity of 18S rRNA in hESC was mapped onto the known 18S rRNA secondary structure. We performed gene-level normalization for 18S rRNA for each cell. The secondary structure image of 18S rRNA is adopted from RiboVision. The red and orange bases indicate high and medium reactive bases respectively from sc-SPORT. **b**, Boxplot showing the distribution of reactivities from bases that are base-paired between adjacent base pairs (left), paired in terminal base pair (middle), and unpaired regions (right) along 18S rRNA. The boxplots show the means and 25th to 75th percentile inter-quartile range, the bars show the range from 5^th^ to 95^th^ percentile. *P*-values were calculated using a two-sided Mann-Whitney U test. The numbers of nucleotides are as shown. **c**, *Left*, Line plot showing the structure reactivities of 18S rRNA from bases 50 to 290, in bulk, 100 cells, 10 cells, and single cells (from top to bottom). The error bars represent the standard error. *Right*, Footprinting gel of 18 s rRNA using NAI-N3. We are showing DMSO-treated RNA (lane 2), and NAI-N3-treated RNA (lane 3). lane 1 (U ladder) shows the base position of Us. The cell number for each condition is labeled. N = 3 independent biological replicates. **d**, Zoomed-in version of a region of 18S rRNA with hESC single-cell pseudobulk reactivity mapped onto the structure. The red and orange bases indicate high and medium reactive bases respectively from sc-SPORT. Blue arrows indicate single-stranded bases from SAFA footprinting data in A. **e**, Scatterplots showing the Spearman correlation of the SHAPE-reactivity 18S rRNA (50–1700nt) in 100 cells, 10 cells, and single cells versus its SHAPE-reactivity in bulk cells. The mutational rate of bases <0.0002 were filtered out. **f**, The AUC-ROC curve of the pseudobulk reactivities of the Tetrahymena ribozyme that is transfected into HEK293T cells. Source data

To show that sc-SPORT can capture RNA structure information accurately, we calculated the area under the curve–receiver operating characteristics (AUC–ROC) of 18S ribosomal RNA in bulk and single cells. We observed a high AUC–ROC of 18S rRNA in single-cell pseudobulk (AUC–ROC of 0.74) and bulk cells (AUC–ROC of 0.72; Fig. 2b). Moreover, the identified single-stranded bases along 18S rRNA are accurate (Extended Data Fig. 4a,b) and consistent with low-throughput footprinting data (Extended Data Fig. 4c,d). We observed an AUC–ROC of 0.6–0.71 for 18S rRNA in single cells (Fig. 2c), indicating that we captured both known single-stranded regions, as well as intracellular variability along 18S rRNA. To show the reliability of sc-SPORT in mapping RNA structures in different numbers of cells, we performed RNA structure probing using millions, 100, 10 and single hESCs as starting material. RNA structure probing data of 18S rRNA showed highly consistent reactivity, regardless of the starting number of cells (Fig. 2d and Extended Data Fig. 4e), indicating that sc-SPORT is reliable. As an additional control, we transfected Tetrahymena ribozyme RNA into HEK293T cells and calculated its single-cell SHAPE reactivity. We observed high AUC–ROC values of 0.74–0.83 in single cells and pseudobulk for the Tetrahymena ribozyme (Fig. 2e and Extended Data Fig. 4f), confirming the robustness of our method.

**Extended Data Fig. 5:**
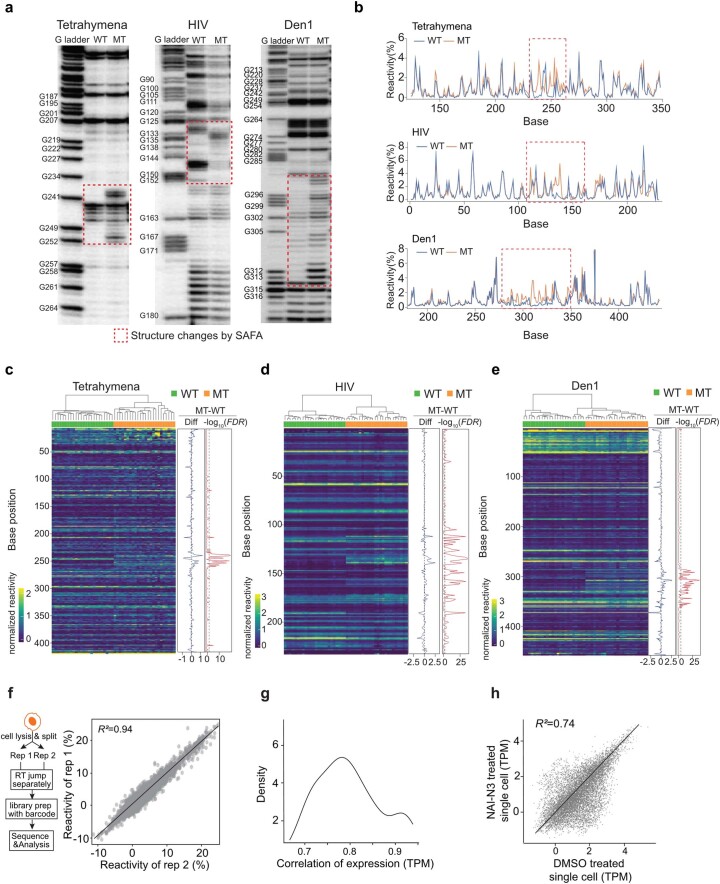
sc-SPORT can accurately identify transfected RNA structures. **a**, SAFA gels showing the reverse transcription stoppage sites along the Tetrahymena ribozyme (left), HIV RNA (middle), and Den1 3’UTR (right). We show NAI-N3 reactivities in the wildtype (WT, lane 2), and NAI-N3 reactivities in the mutant RNA (MT lane 3). lane 1 (G ladder) of each gel shows the base position of Gs. The red dashed boxes show the structural differences between the wildtype and mutated regions. N = 3 independent biological replicates. **b**, Line plots showing the pseudobulk reactivity of transfected wildtype (WT, blue) and mutant (MT, orange) RNAs inside single cells. Top: Tetrahymena ribozyme, middle: HIV RNA, bottom: dengue 3’UTR RNA (Den1). **c**–**e** Unsupervised clustering based on the structure reactivity at each base of Tetrahymena (**c**), HIV (**d**), and Den1 (**e**) in single cells. The dendrogram was calculated with Euclidean distances and the ward method. *Left*: The heatmap showed the reactivity of transfected WT and mutant tetrahymena RNA(**c**), HIV RNA(**d**), and Den1 (**e**) in single cells. Each column is a cell and each row is the reactivity for that base. *Middle*: Line plot showing the difference in average reactivity between wildtype and mutated transcript. *Right*: Line plot showing the significance of structure difference between WT and MT reactivities, calculated as -log_10_(FDR) using a two-sided Student’s T-test. The black dashed line indicates *p* = 0.05. **f**, The schematic (left) shows how the RNAs from a single cell were separated into two populations for downstream library preparation. The scatter plot shows the correlation in reactivity from two technical replicates of an NAI-N3-modified single cell. **g**, Density plot showing the distribution of the correlation between TPM per transcript from NAI-N3 treated single cells and from the pseudobulk of DMSO treated cells. **h**, Scatterplot showing the correlation between gene expression (TPM) for transcripts from a selected NAI-N3 treated cell and gene expression (TPM) for transcripts from the pseudobulk of DMSO treated cells. We observed a high correlation of *R*^2^ = 0.74. Source data

In addition to detecting RNA structure information in a single cell, we tested whether we could also identify structural differences in RNAs between individual cells. To do this, we introduced three different RNAs (Tetrahymena ribozyme, Den1 and human immunodeficiency virus (HIV) RNA) into a population of HEK293T cells and separately introduced a structure mutant (MT) version of these RNAs, with a few disrupted paired bases, into another population of cells (Fig. 2f and Methods). After performing sc-SPORT, we first confirmed that we can observe structural differences between the wildtype (WT) and MT RNAs at the level of pseudobulk (Fig. 2f and Extended Data Fig. 5a,b). We then confirmed that we can cluster single cells using RNA structure differences (Extended Data Fig. 5c–e), suggesting that RNA structure information can be used to separate cellular populations (Fig. 2g–i).

To further show that sc-SPORT is highly reproducible, we performed a control whereby we treated a single cell with NAI-N3 and split the modified cellular RNAs into two pools before performing the library preparation for each pool. We observed a very high correlation in SHAPE reactivity (*R*^2^ = 0.94) between the two technical replicates from the same cell, indicating that sc-SPORT is highly reproducible (Extended Data Fig. 5f). Last, we observed that summing reads along a transcript from sc-SPORT correlates well with gene expression obtained from the pseudobulk of untreated cells, indicating that our sc-SPORT data captures both RNA expression information and RNA structural information accurately in a single cell (Extended Data Fig. 5g,h).

### RNAs can fold into different structures in individual cells

**Extended Data Fig. 6:**
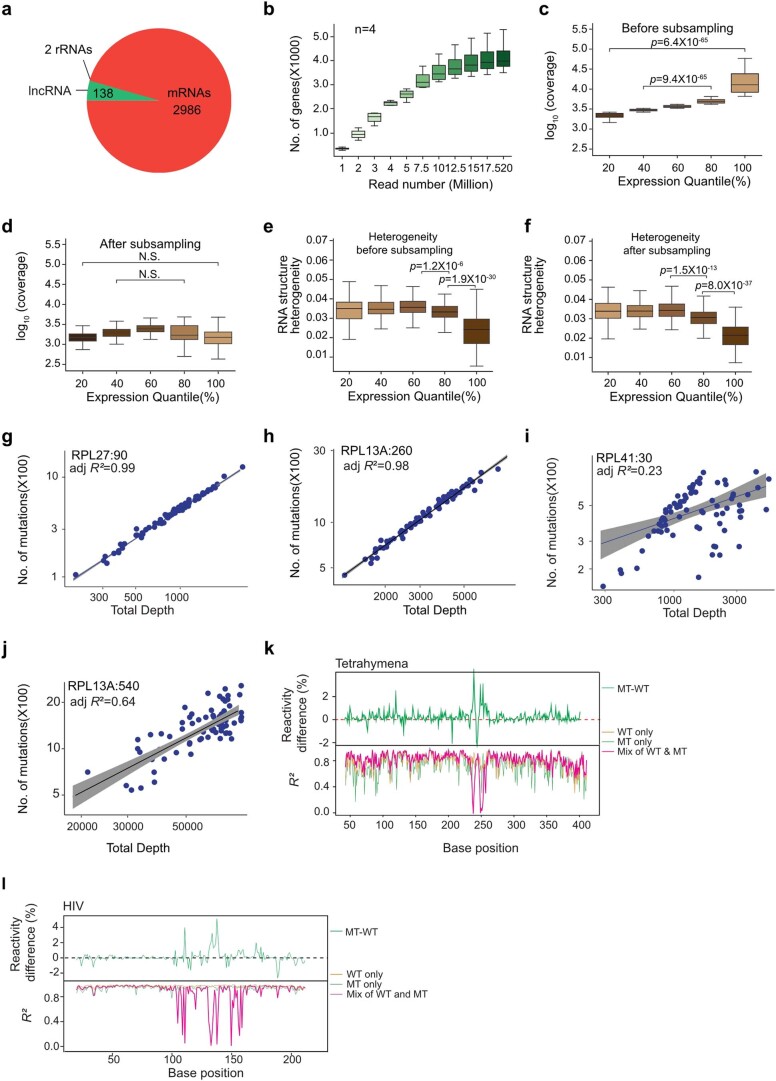
sc-SPORT identifies structurally heterogeneous regions. **a**, Pie chart showing the average number of transcripts that we can detect structure information in single hESC cells. **b**, Boxplot showing the distribution of the number of detected genes at various depths of sequencing reads, from sub-sampling. Each boxplot represents 4 replicates of subsampling. **c, d**, Boxplots showing the distribution of read coverage per nucleotide in genes that are present in different abundance groups in hESCs before (**c**) and after subsampling (**d**). The *p*-values were calculated by a two-sided Mann-Whitney U test. We subsampled the reads from the genes of the two highest abundance groups so that their coverage is similar to the genes of the other three abundance groups. **e, f**, Boxplots showing the distribution of structural heterogeneity for transcripts before (**e**) and after (**f**) subsampling of their abundance. The *p*-values were calculated by a two-sided Mann-Whitney U test. The box plots show the means and 25th to 75th percentile inter-quartile range, the bars show the range from 5^th^ to 95^th^ percentile. The numbers of genes in each percentile are 194, 193, 193, 193, 193 respectively. **g-j**, Scatter plots of the total read count and the total mutant read count for two structurally homogeneous windows in RPL27 (ribosomal protein L27, pos=90, **g**), and RBP13A (ribosomal protein L13a, pos=260, **h**), and two structurally heterogeneous windows in RPL41 (pos=30, **i**) and RPL13A (pos=540, **j**). The window-level structural heterogeneity (adjusted *R*^2^ values) is labeled inside each plot. The error bands represent the 95% confidence intervals of linear regression. **k, l**, Top: Line plots showing the difference in SHAPE-reactivity between WT and mutant Tetrahymena (**k**) and HIV (**l**) RNAs. Bottom: Line plots showing the structural heterogeneity values (*R*^2^) for WT, MT, and a mix of WT and MT Tetrahymena ribozyme (**k**) and HIV (**l**). We only observe low *R*^2^ values at structure-changing regions when WT and MT RNAs are mixed, indicating that we are detecting heterogeneity correctly.

We performed two biological replicates of sc-SPORT in hESCs by sequencing 5–10 million reads per cell for 40 cells in each replicate. We obtained RNA reactivities for an average of 3,146 genes, including 2,986 messenger RNAs, 138 long noncoding RNAs and 2 rRNAs in each hESC (Extended Data Fig. 6a). To determine the amount of sequencing needed for single-cell RNA structure determination, we sequenced four single cells to a much higher depth of 20 million reads per cell. The number of transcripts with RNA structure information that we can detect increases linearly with sequencing up to around 10 million reads per cell, after which the transcript number starts to plateau (Extended Data Fig. 6b). At a standard single-cell sequencing depth of around 2 million reads, we can obtain structural information of ∼1,000 genes in a single hESC.

**Fig. 3:**
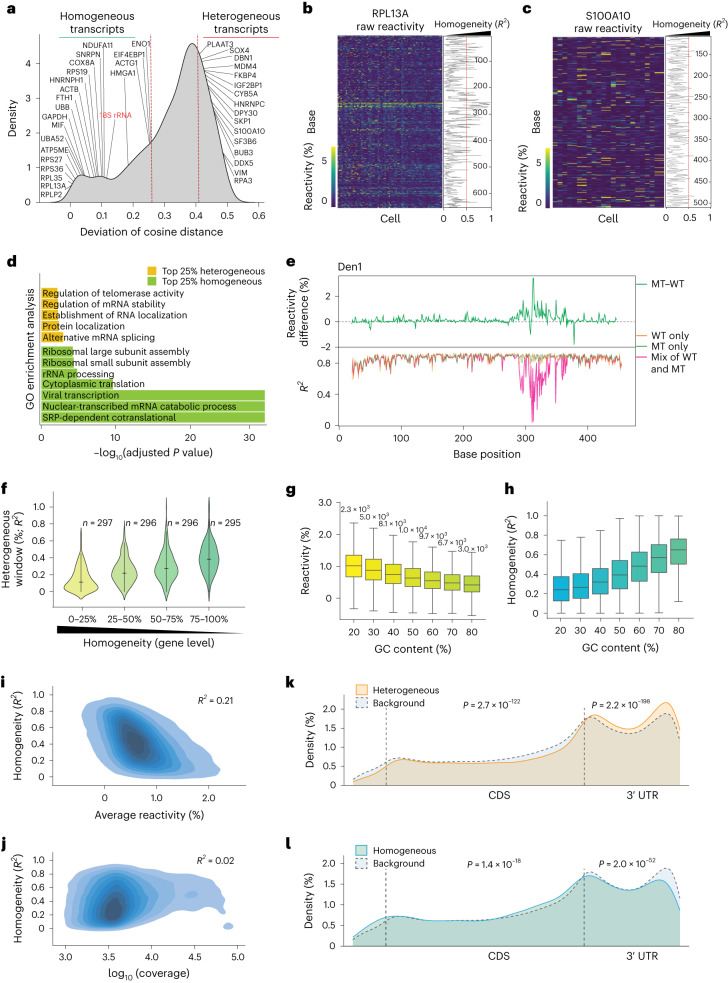
Single-cell RNA structure features in the hESC transcriptome. **a**, A density plot showing the distribution of structural heterogeneity at a transcript level for the hESCs. Red dashed lines indicate 25% percentile (most homogeneous) and 75% percentile (most heterogeneous) of transcripts. The names of selected genes are labeled, and 18S rRNA is labeled in red. **b**,**c**, A heatmap showing per base reactivity in hESCs for a stable (**b**) and variable (**c**) transcript. Each row is a nucleotide along the transcript and each column indicates a cell. The line plot on the right indicates the *R*^2^ value of each nucleotide in the heatmap. **d**, A bar plot showing the enriched GO terms for the most heterogeneous (yellow) and homogeneous (green) transcripts. *P* values were calculated using the Fisher exact test. **e**, Top: a line plot showing the difference in SHAPE reactivity between WT and MT Den1 RNA. Bottom: line plots showing the structural heterogeneity values (*R*^2^) for WT, MT and a mix of WT and MT Den1 RNA. **f**, Violin plots showing the distribution of heterogeneous windows (*R*^2^) in genes with different extents of homogeneity (deviation of cosine distance). The bars in the violin plot represent the median and the interquartile range. The numbers are as shown. **g**, Box plots showing the distribution of the average reactivity of 10 nt windows with increasing GC content. The window numbers of different GC content are as shown. **h**, Box plots showing the distribution of the homogeneity (*R*^2^) of 10 nt windows with increasing GC content. The window numbers of different GC content are as shown in **g**. The box plots show the means and 25th to 75th percentile interquartile range, and the bars show the range from 5th to 95th percentile. **i**, Density plot showing the correlation of homogeneity (*R*^2^) with the average reactivity of 10 nt windows (window no. 56,096). **j**, Density plot showing the correlation between homogeneity (*R*^2^) and read coverages of 10 nt windows. **k**,**l**, A metagene analysis of the distribution of 25% most heterogeneous (**k**) and homogeneous windows (**l**) centered on the start and stop codon of mRNAs. The background line indicates all detected windows*.* The *P* value was calculated by a one-sided hypergeometric test.

One of the key questions in RNA structure is whether the same RNA can form different structures in individual cells. To address this question, we calculated RNA structural heterogeneity along transcripts in each cell (Methods) and binned the transcripts into different quantiles of structure variability (Fig. 3a and Supplementary Table 2). As structurally homogeneous transcripts show little variation in their reactivity, and heterogeneous transcripts show more variation in their reactivity across single cells (Fig. 3b,c), we can identify structurally homogeneous and heterogeneous transcripts in hESC single cells. As expected, 18S rRNA is one of the most structurally homogeneous RNAs among all detected transcripts (Fig. 3a), agreeing with the importance of its structure for its function. Additionally, we observed that many mRNAs involved in translation, including ribosomal protein mRNAs and translation elongation factors, are more structurally homogeneous (Fig. 3a). Overall, Gene Ontology (GO) term enrichments of highly homogeneous genes are associated with ribosomal assembly, rRNA processing and translation-related biological processes (Fig. 3d), suggesting that transcripts associated with key cellular processes are tightly regulated at the structure level. In addition, the GO term enrichments of highly heterogeneous genes are associated with mRNA stability, the establishment of RNA localization, protein localization and alternative mRNA splicing-related biological processes (Fig. 3d), linking RNA structure variability to gene regulation.

As structurally homogeneous transcripts also tend to be highly expressed, we confirmed that our calculated homogeneity is not due to our ability to calculate RNA reactivities more accurately in abundant transcripts, by subsampling the abundant transcripts to the median depth of all our detectable transcripts (Extended Data Fig. 6c,d). Subsampling of the abundant transcripts showed that they remain more structurally homogeneous than the less abundant transcripts, indicating that their homogeneity is not due to experimental limitations (Extended Data Fig. 6e,f).

We observed that different windows along a transcript can display various extents of structural heterogeneity between individual cells (Extended Data Fig. 6g–j). To identify structurally homogeneous/heterogenous regions, we calculated the variation in reactivity in 10 nt windows using linear regression in single cells (Methods). Indeed, RNA regions in transfected RNAs with structure mutations show lower *R*^2^ values and exhibit higher variability across single cells (Fig. 3e and Extended Data Fig. 6k,l). Additionally, we show that structurally homogeneous transcripts, determined by variation of cosine distance, show a larger proportion of homogenous windows using *R*^2^ (Fig. 3f), confirming that our heterogeneity measurements are accurate.

To understand the properties underlying homogeneous or heterogeneous windows in the human transcriptome, we tested the correlation of these windows with SHAPE reactivity, GC content and abundance of that window (Fig. 3g–j). We confirmed that windows with low reactivity are correlated with increased GC content (Fig. 3g). Additionally, we observed that homogeneous windows are associated with higher GC content (Fig. 3h), have lower reactivity (Fig. 3i) and are independent of the abundance of the window (Fig. 3j). We next examined the location of these structurally heterogeneous/homogeneous windows in mRNAs by calculating their frequencies in the coding region (CDS), 5′ and 3′ UTRs. We observed that 3′ UTRs are significantly enriched for heterogeneous windows as compared with CDS, suggesting that they are more structurally variable in single cells (Fig. 3k,l).

### Structural heterogeneity can better inform RBP binding

**Fig. 4:**
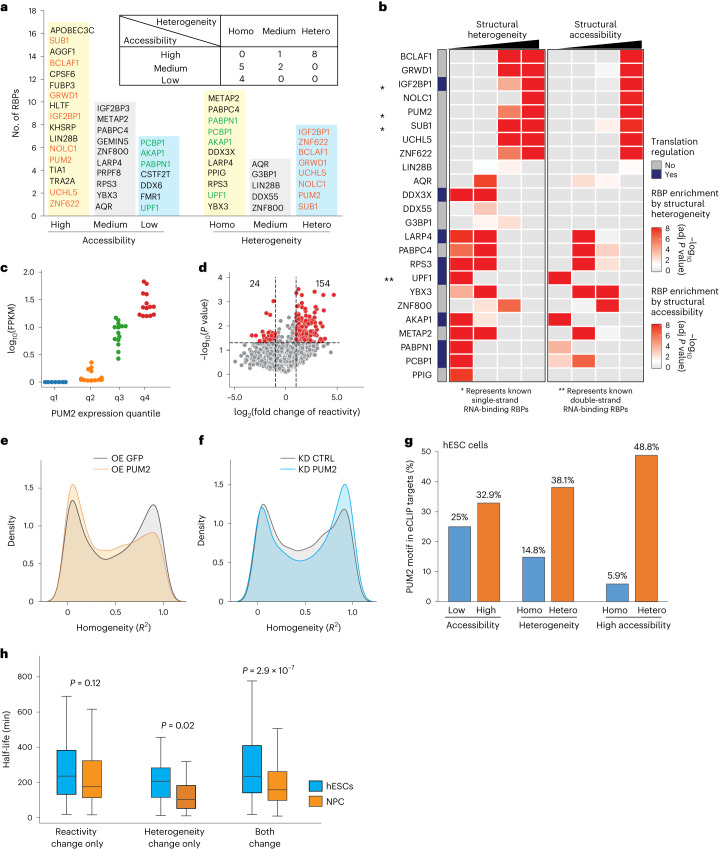
RBPs regulate structure heterogeneity in single cells. **a**, A diagram illustrating the names of RBPs that are enriched in different accessible regions and heterogeneous regions. The table shows the number of RBPs shared in windows with different heterogeneity and accessibility levels. Homo, homogeneous; hetero, heterogeneous. **b**, A heatmap showing the −log_10_-adjusted *P* value (adj *P* value) of enrichment, for selected RBPs, in windows with different heterogeneity (left) and reactivity levels (right). The *P* values were calculated by a one-sided hypergeometric test. RBPs involved in translation are shown in purple on the left. * or ** represents known RBPs that bind single-strand or double-strand RNA, respectively. **c**, A swarm plot showing PUM2 expression levels in single cells. Cells are separated according to four different quantiles of PUM2 abundance (0–25% (blue), 25–50% (orange), 50–75% (green) and 75–100% (red)). The *y* axis indicates RNA expression levels of PUM2 in each cell. **d**, A volcano plot showing changes in structure reactivities between low and high PUM2 expressed cells for windows located within PUM2-binding regions. The *x* axis shows the log_2_-fold change of their reactivities in q1 and q4 quantiles. The *P* values were calculated using a two-sided Student’s *t*-test. **e**, Density plots showing the distribution of PUM2-binding motif heterogeneity and 50 nt flanking region in green fluorescent protein (GFP) overexpressed (OE, gray) and PUM2 overexpressed (yellow) hESCs. **f**, Density plots showing the distribution of PUM2-binding motif heterogeneity and 50 nt flanking region in control (CTRL) knocked-down (KD, gray) and PUM2 knocked-down (blue) hESCs. **g**, Bar plots showing the percentage of PUM2 motifs present in eCLIP experiments in high or low accessible regions (left), homogeneous or heterogeneous regions (middle) and accessible regions that are homogeneous or heterogeneous (right) in hESCs. **h**, A box plot showing the distribution of RNA half-life in hESC and neuronal precursor cell (NPC) (Supplementary Table 5) for transcripts that show reactivity changes (left, gene no. 127), heterogeneity changes (middle, gene no. 34) and both reactivity and heterogeneity changes (right, gene no. 145) upon overexpression of PUM2 in hESCs. The *P* values were calculated using a two-sided Mann–Whitney *U* test. The box plots show the means and 25th to 75th percentile interquartile range, and the bars show the range from the 5th to 95th percentile.

**Extended Data Fig. 7:**
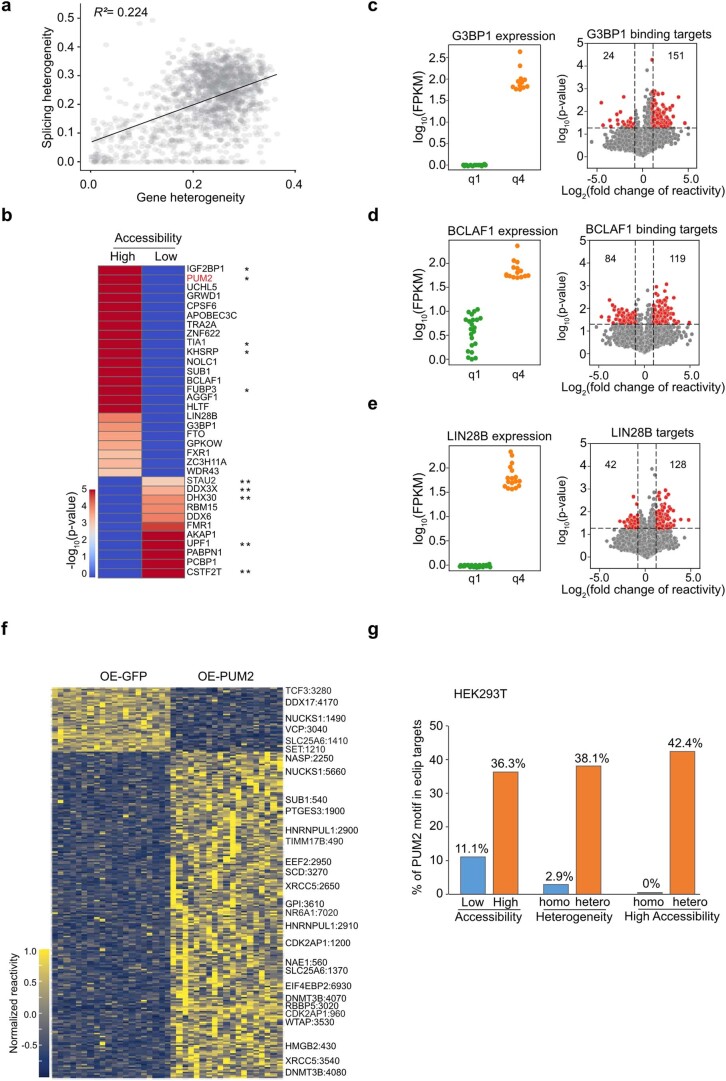
RNA structural heterogeneity is associated with RBP binding. **a**, Scatter plot showing the correlation between changes in splicing heterogeneity and structural heterogeneity in hESC single cells. The splicing heterogeneity was defined as the Shannon entropy of isoform proportions in hESC single cells, and structure heterogeneity was measured as the deviation of cosine distance for each gene. **b**, Heatmap showing enriched RBPs at more accessible and less accessible regions along the hESC transcriptome. The more and less accessible regions are calculated from the pseudobulk reactivities in the hESC transcriptome. *P*-value is calculated using a one-sided Fisher’s Exact Test. * represents Known RBPs that bind to single-stranded target regions and ** represents known RBPs that bind to double-stranded target regions. **c-e**, *Left*, Swarm plot showing the G3BP1 (**c**), BCLAF1 (**d**), and LIN28B (**e**) expression levels in cells that express the first and last quantiles of the RBP. *Right*, Volcano plot showing the structure reactivity changes from low-RBP expressed cells and high-RBP expressed cells for windows located within G3BP1 (**c**), BCLAF1 (**d**), and LIN28B (**e**) binding regions. The binding regions were determined from eCLIP peaks from ENCODE. *P*-values were calculated by a two-sided Wald test in DESeq2. **f**, Heatmap shows the structural differences between control and PUM2 overexpressed cells. The significant structural differences were calculated using Student’s T-Test between RNA reactivity in 20 control cells (GFP) versus 20 PUM2 overexpressed cells. **g**, Barplots showing the percentage of PUM2 binding sites from eCLIP data that exist in more accessible or less accessible regions (left), homogeneous or heterogeneous regions (middle), and more accessible regions that are structurally homogeneous or heterogeneous (right) in HEK293T cells.

Human 3′ UTRs undergo extensive processing^20^, including alternative splicing, alternative polyA usage, RNA modifications and RNA binding protein (RBP) binding^21–23^. To evaluate the effect of alternative splicing on RNA structure heterogeneity, we asked whether transcripts with high structure heterogeneity also show large changes in the relative proportions of transcript isoforms in single cells. We observed a weak correlation between the two (Extended Data Fig. 7a), indicating that alternative splicing is not a major contributor to structural heterogeneity. To identify other regulators that could modulate structure heterogeneity, we asked whether differentially heterogeneous regions could be enriched for RBPs. We analyzed the data using an enhanced crosslinking and immunoprecipitation (eCLIP) dataset from ENCODE^24^ and confirmed that more accessible regions are enriched for single-strand specific RBPs such as pumilio homolog 2 (PUM2) (ref. ^25^), insulin-like growth factor 2 mRNA binding protein 1 (IGF2BP1) (ref. ^26^), SUB1 regulator of transcription (SUB1) (ref. ^27^), lin-28 homolog B (Lin28B) (ref. ^28^) and G3BP stress granule assembly factor 1 (G3BP1) (ref. ^29^) (Fig. 4a and Extended Data Fig. 7b). We also confirmed that less accessible regions are enriched for double-strand specific RBPs such as staufen double-stranded RNA binding protein 2 (STAU2) (ref. ^30^), DEAD-box helicase 3 X-linked (DDX3X) (ref. ^31^), UPF1 RNA helicase and ATPase (UPF1)^32^, and DEAD-box helicase 55 (DDX55) (ref. ^33^) (Fig. 4a and Extended Data Fig. 7b), confirming that our single-cell data captures known patterns from bulk cells.

Calculating the enrichments of RBPs on heterogeneous and homogeneous regions identified 11 and 8 RBPs enriched in homogeneous and heterogeneous regions, respectively (Fig. 4a). RBPs enriched for homogeneous/heterogeneous RNA regions are generally associated with low and high reactivity regions, respectively (Fig. 4a,b). On the basis of our enrichments, we hypothesize that RBPs without clear structural preference in the literature, including A-kinase anchoring protein 1 (AKAP1), poly(rC) binding protein 1 (PCBP1) and poly(A) binding protein nuclear 1 (PABPN1), are likely to bind to low reactivity regions, and RBPs such as nucleolar and coiled-body phosphoprotein 1 (NOLC1), BCL2-associated transcription factor 1 (BCLAF1), zinc finger protein 622 (ZNF622), glutamate-rich WD repeat containing 1 (GRWD1) and ubiquitin C-terminal hydrolase L5 (UCHL5) are likely to bind to high reactivity regions on their targets. Additionally, we observed that RBPs that are enriched for structurally homogeneous substrates are more strongly associated with translation than RBPs that are enriched for structurally heterogeneous substrates (Fig. 4b). This coincides with our observation that structurally homogeneous transcripts are enriched for translation processes, suggesting potentially coordinated regulation inside cells (Fig. 3d).

To show that RBP expression could regulate RNA structure in single cells, we focused our analysis on PUM2, which encodes a single-strand specific RBP with a highly conserved binding motif^25^. Our bulk structure analysis has shown that PUM2 binding to its substrates results in increased accessibility of its substrates^12^. To ask whether different levels of PUM2 in single cells can result in different reactivities in the same cell, we binned cells according to their respective PUM2 levels and calculated the reactivity of PUM2 target regions in each population of cells (Fig. 4c). We observed that PUM2 substrates present in cells with high PUM2 levels show an increase in accessibility as compared with PUM2 substrates in cells with low PUM2 levels (Fig. 4d), supporting that PUM2 levels impact structure accessibility in single cells. We additionally calculated the substrate reactivity of other RBPs in cells with high and low levels of the RBP and confirmed that RBP is an important class of structure regulators in single cells (Extended Data Fig. 7c–e).

To confirm that PUM2 regulates structural heterogeneity, we overexpressed or knocked down PUM2 in hESCs and determined the structural accessibility of PUM2 targets before and after^24^. Upon PUM2 overexpression, we observed that 69.9% of its targets showed an increase in accessibility and heterogeneity in single cells (Fig. 4e and Extended Data Fig. 7f). PUM2 knockdown showed an inverse effect from its overexpression, with 64.2% of PUM2’s targets becoming less accessible and more homogeneous (Fig. 4f). To determine whether structural heterogeneity can complement structural accessibility to better predict RBP binding, we sorted PUM2-binding motifs into three classes according to whether they have high or low reactivities, are structurally homo/heterogeneous or both. We then calculated the proportion of PUM2 binding, based on eCLIP data, in each category. As expected, accessible PUM2 motifs have a higher proportion of PUM2 eCLIP binding sites (32.9%) than inaccessible PUM2 motifs (25%; Fig. 4g). Structurally heterogeneous PUM2 motifs are also occupied by real PUM2-binding sites more frequently than homogeneous motifs (38.1% versus 14.8%; Fig. 4g). Importantly, PUM2 motifs that are both accessible and structurally heterogeneous contained the highest proportion of real PUM2-binding sites (48.8%; Fig. 4g), while motifs that are accessible and yet structurally homogeneous have the lowest percentage of real PUM2-binding sites (5.9%). These data indicate that structural heterogeneity information can be used to better predict real PUM2-binding sites and eliminate false PUM2-binding sites. To show that this observation is not limited to hESC, we repeated single-cell structure probing in a different cell type and observed a similar trend in HEK293T cells (Extended Data Fig. 7g).

**Extended Data Fig. 8:**
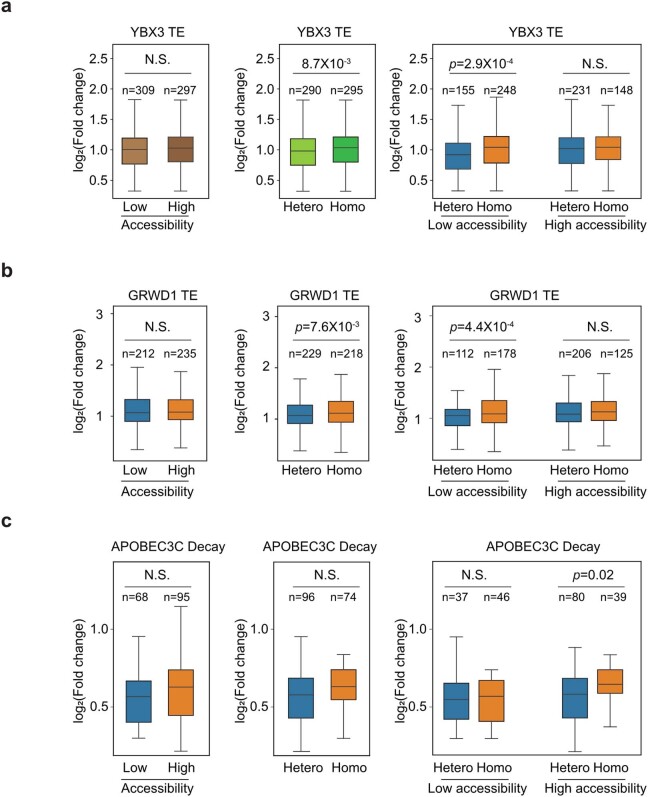
RNA structural heterogeneity impacts gene regulation. **a, b**, Boxplots showing the distribution of change in translation efficiency between YBX3 (**a**) or GRWD1 (**b**) target transcripts in hESC and NPCs when we separate YBX3 (**a**) or GRWD1 (**b**) target windows according to whether they are less or more accessible (left), homo or heterogeneous (middle), or both (right) in hESC. **c**, Boxplots showing the distribution of change in half-life between APOBEC3C target transcripts in hESC and NPCs when we separate APOBEC3C target windows according to whether they are less or more accessible (left), homo or heterogeneous (middle), or both (right) in hESC. The boxplots show the means and 25th to 75th percentile inter-quartile range, the bars show the range from 5^th^ to 95^th^ percentile. The numbers of genes in each boxplot are as shown. All the *p*-values were calculated by the two-sided Mann-Whitney U test in this figure.

PUM2 protein levels are increased during neuronal differentiation to regulate translation and decay^12^. To determine whether structural heterogeneity can provide additional insights into PUM2 gene regulation, we identified transcripts that showed changes in accessibility, structural heterogeneity or both, upon an increase in PUM2 protein levels, and determined their half-lives in hESCs and neuronal precursor cells (NPCs). Interestingly, while transcripts with changes in accessibility in the presence of PUM2 showed decreased half-lives in NPCs, this trend becomes stronger in transcripts with changes in heterogeneity. Transcripts with both heterogeneity and reactivity changes in the presence of PUM2 showed the largest half-life difference between hESC and NPC (Fig. 4h), indicating that both structural heterogeneity and reactivity can impact gene regulation. In addition to PUM2, we identified three other RBPs (Y-box-binding protein 2 (YBX2), glutamate-rich WD repeat containing 1 (GRWD1) and apolipoprotein B mRNA editing enzyme catalytic subunit 3C (APOBEC3C)) whereby the structural heterogeneity of their targets impacts gene regulation, either at the level of translation or decay (Extended Data Fig. 8a–c), indicating that structural heterogeneity could be an important feature in RBP regulation.

### RNA structures vary in single cells during differentiation

**Fig. 5:**
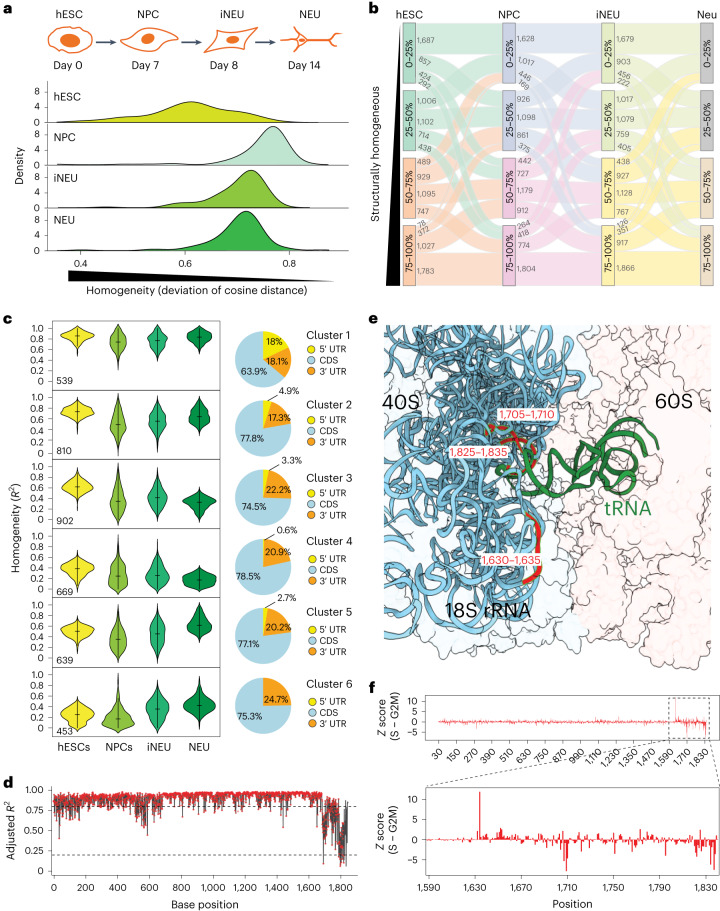
Sc-SPORT identifies single-cell RNA structure dynamics. **a,** Top: a schematic showing the timelines and cellular identities during neuronal differentiation in this study. Bottom: density plots showing the distribution of gene-level heterogeneity in single cells in each stage from hESC (day 0) to NPC (day 7), iNEU (day 8) and NEU (day 14, top to bottom). Heterogeneity is calculated using the deviation of cosine distance. Only shared genes across four stages were included in the distribution. **b**, Sankey plots showing the changes in window-level heterogeneity during the neuronal differentiation process. To simplify, shared windows of four stages are grouped into four quartiles according to their heterogeneity values in each stage. **c**, *K*-means clustering with dynamic time warping by their heterogeneity across the four stages of neuronal differentiation. We identified six clusters that show different patterns of heterogeneity during differentiation. Left: violin plots show the distribution of structural heterogeneity in the four stages from left to right for each cluster. The bars in the violin plot represent the median and the interquartile range of heterogeneity. The numbers of windows in each cluster are as shown. Right: pie charts show the distribution of the windows present in 5′ UTR, CDS and 3′ UTR of mRNAs. **d**, Line plots showing per-nucleotide heterogeneity (*R*^2^) of 18S rRNA in 76 hESC cells. **e**, Zoomed-in view showing the location of our identified heterogeneous region in 18S rRNA in its three-dimensional model. The three-dimensional structure is obtained from PDB (PDB ID: 4v6x). We have colored the transfer RNA in green and our changing regions in red. **f**, A bar plot showing the *∆*mutation rates of 18S rRNA between single cells in the S phase and G2/M phase (top). *∆*Mutation rates are calculated by subtracting the pseudobulk reactivity of cells in the S phase and the G2/M phase. The black dashed box shows the heterogeneous region (1,590–1,830) and is zoomed-in as below.

**Extended Data Fig. 9:**
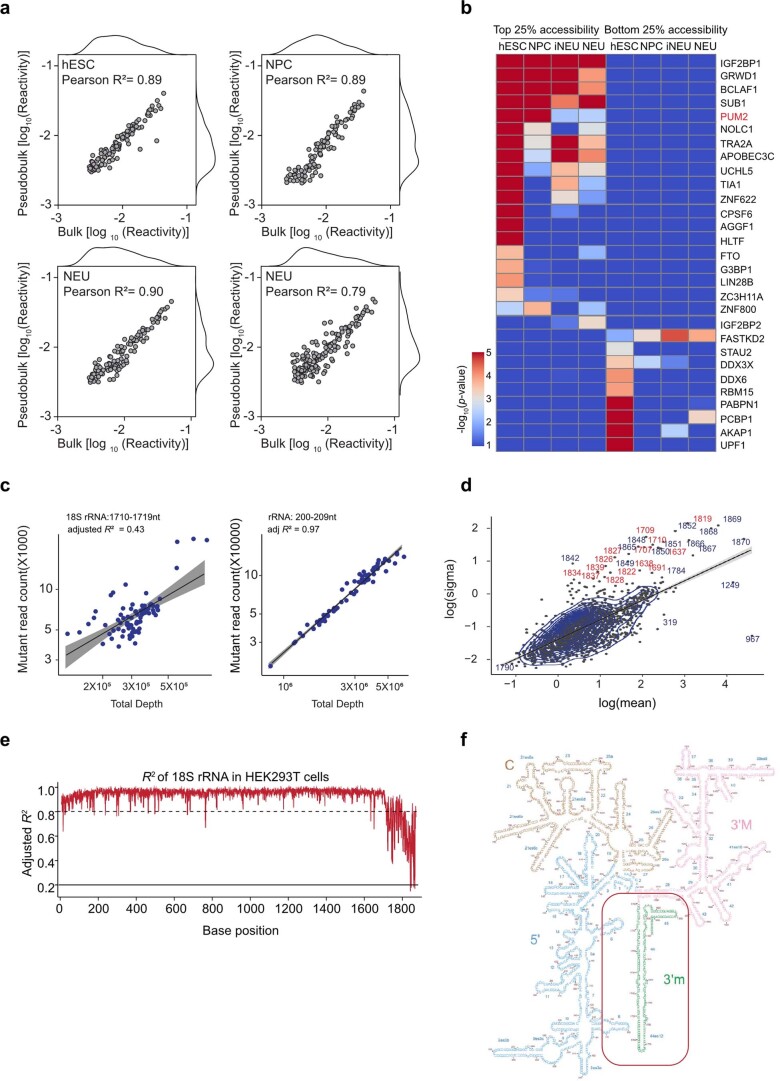
sc-SPORT identifies structural dynamics in single cells. **a**, Scatter plots showing the correlation between structure reactivities of 18S rRNA (50–1700 nt) pseudobulk and bulk data for ESC, NPC, iNEU, and NEU. The bases with negative reactivities were filtered out. **b**, Heatmap showing the Phred scores of RBP enrichment in the 25% most accessible and 25% least accessible windows in each stage. The *P*-value is calculated using Fisher’s Exact Test. **c**, Scatter plots showing the total coverage depth (X-axis) and total mutant read counts (Y-axis) for a structurally heterogeneous window on position 1710 (*left*) and a structurally homogeneous window on position 200 (*right*), on 18S rRNA, in hESCs. The error bar represents the 95% confidence intervals of linear regression. **d**, Scatter plot showing the mean mutation rate and standard deviation for each nucleotide of 18S rRNA from hESC cells. The nucleotides which are far away from the regression lines are labeled with their positions. The positions that belong to Helix 44 and 45 are highlighted in red. The error bar represents the 95% confidence intervals of linear regression. **e**, The heterogeneity (*R*^2^) of each nucleotide on 18S rRNA in HEK293T cells. **f**, The secondary structure diagram of 18S rRNA from Ribovision. Helix 44 and 45 are labeled with a red rectangle box.

Neuronal differentiation is a complex process with extensive posttranscriptional gene regulation. We have previously observed extensive structural changes as hESCs differentiate into different cell stages^12^. However, whether all the cells in a population changed structure or only a subset of cells changed structure during differentiation remains unknown. To study RNA structure changes at a single-cell level during neuronal differentiation, we performed two biological replicates of sc-SPORT on 312 individual cells at different stages of neuronal differentiation: hESC, NPC (7 days post-differentiation), immature neurons (iNeu, 8 days post-differentiation) and early neurons (NEU, 14 days post-differentiation) (Fig. 5a). Sc-SPORT pseudobulk reactivities of 18S rRNA showed a good correlation with bulk cell reactivities in each cellular stage (Extended Data Fig. 9a), indicating that our data are of good quality. Additionally, we confirmed that regions with high reactivity in the pseudobulk of all four stages are enriched for single-strand specific RBPs, agreeing with these regions being unpaired for RBP binding (Extended Data Fig. 9b).

Globally, we observed that transcripts from hESCs are more structurally homogeneous than transcripts from single cells in other differentiated states (Fig. 5a), with transcripts from individual cells in the NPC stage showing the largest structural heterogeneity. This agrees with the observation that NPCs are the most morphologically and biologically diverse among the four cell types^34^. We observed that 7,373, 7,331 and 7,288 windows demonstrated RNA structure heterogeneity changes, with 52.9%, 48.4% and 51.8% of the windows becoming more homogeneous, as hESC differentiated into NPCs, NPCs differentiated into iNeu and iNeu differentiated into neurons, respectively (Fig. 5b). Unsupervised clustering of RNA windows based on their structural heterogeneity identified six clusters (Fig. 5c and Supplementary Table 3), including consistently homogeneous regions (cluster 1), increasingly heterogeneous (clusters 3 and 4), increasingly homogeneous (clusters 5 and 6) and heterogeneous regions in NPCs (cluster 2). Interestingly, consistently homogeneous RNA regions (cluster 1) are enriched in the 5′ UTRs of mRNAs (Fig. 5c), suggesting that their structures are conserved during neuronal differentiation.

While 18S rRNA is one of the most structurally homogeneous transcripts in our dataset, we identified a highly heterogeneous region near the 3′ end, which corresponds to helix 44 and 45 in single cells (Fig. 5d and Extended Data Fig. 9c). We used an orthogonal way to calculate heterogeneity and confirmed that these regions are indeed the most heterogeneous along 18S rRNA in both hESCs and HEK293T cells (Extended Data Fig. 9d,e). A deeper examination of these heterogeneous bases revealed that they are located at the mRNA tunnel in the 40S subunit (Fig. 5e and Extended Data Fig. 9f). As the mRNA tunnel is involved in translation initiation, we asked whether cells with different rates of translation could show differences in the reactivity of these bases. As we lack direct translation data in the same cells, we utilized the information that cells at different stages of the cell cycle exhibit different levels of translation^35^. As such, we separated hESCs into whether they are in G2/M or S phases based on their cell cycle expression markers and identified 40 and 35 cells in G2/M and S phases, respectively. We observed that the pseudobulk reactivity of the 18S rRNA in the G2/M and S phases is indeed different (Fig. 5f), supporting the hypothesis that structural dynamics in helix 44 is associated with translation. These data demonstrate that sc-SPORT can identify regional regions of structural heterogeneity in the transcriptome in single cells.

### Structure heterogeneity can separate cell populations

**Fig. 6:**
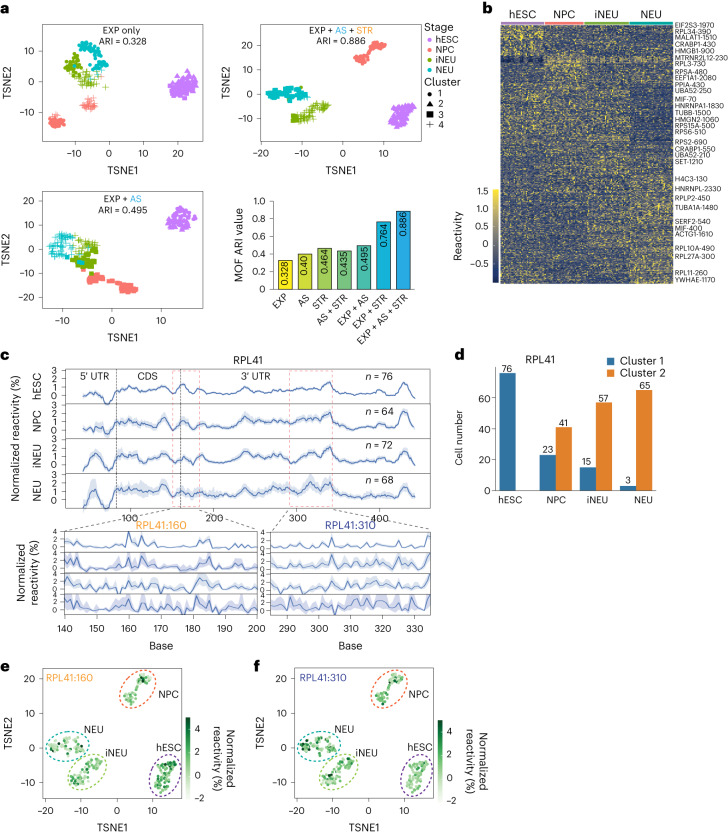
Structures enable better delineation of cellular identity. **a**, Scatter plots showing the MOFA clustering using (1) RNA expression only (top left), (2) RNA expression and alternative splicing (bottom left) and (3) RNA expression, alternative splicing and structural heterogeneity (top right). Bottom right: ARI for the MOFA clustering results using different datasets as input. **b**, A heatmap showing reactivity of stage-specific structures across neuronal differentiation. Each row shows the reactivity of a 10 nt window, and each column is the reactivity for a single cell. Color scale: *z*-score transformed reactivity by row. Selected genes with structure-changing windows were labeled at the right of the heatmap. **c**, Top: pseudobulk reactivity of RPL41 in each stage of neuronal differentiation. The red dashed boxes indicate two structure-changing windows during neuronal differentiation. Single-nucleotide reactivity was smoothed using a 10 nt sliding window. Bottom: single-nucleotide reactivity is shown in the zoomed-in versions of the structure-changing regions. The blue curves were the average reactivity of cells in each stage. The light-blue shading indicates the standard error for each nucleotide. The cell number for each time point is labeled. **d**, Unsupervised clustering of RPL41 RNA structures in single cells during the neuronal differentiation process. The bar plot shows the number of cells that contain cluster 1 and cluster 2 in hESCs and different stages of neuronal differentiation. **e**,**f**, T-distributed stochastic neighbor embedding (TSNE) plot showing the distribution of reactivity of RPL41:160 (**e**) and RPL41:310 (**f**) in all single hESC and differentiated single cells.

Refining cellular identities helps to better understand cellular functions and cell fate trajectories. Traditionally, transcriptome information including gene expression, alternative splicing and polyA usage can be used to delineate cellular populations^36^. To further investigate the role of RNA structural heterogeneity in defining cellular states, we employed multiomics factor analysis 2 (MOFA2) to jointly analyze the RNA expression, alternative splicing and structural heterogeneity to identify the main sources of variation from the data types in an unsupervised manner. We observed that the addition of structural heterogeneity to gene expression and alternative splicing information could greatly increase the adjusted-rank index (ARI) by ∼79% (from 0.495 to 0.886; Fig. 6a), enabling us to better separate NPCs, iNeu and Neu cells. As these cell states could not be separated by gene expression and alternative splicing information alone, our data suggest that single-cell structure information can improve cell clustering or better define cellular populations.

**Extended Data Fig. 10:**
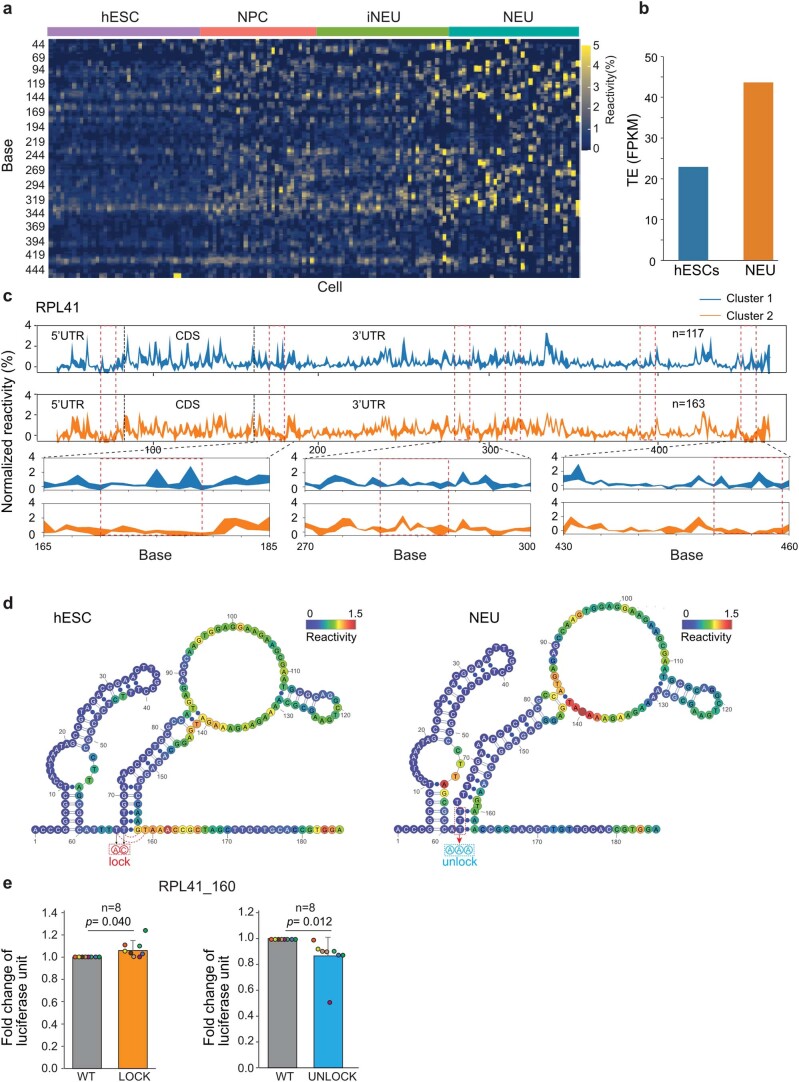
RPL41 shows structural heterogeneity. **a**, Heatmap showing the reactivity of RPL41 during neuronal differentiation. Each row indicates the reactivity for the base, after 10nt smoothing, along the gene, and each column indicates the reactivity in a single cell in each stage. **b**, Translation efficiency of RPL41 in hESC cells (blue) and NEU cells (orange). **c**, RPL41 forms two clusters based on its reactivity. Top, pseudobulk reactivity of RPL41 in each cluster. The cells were clustered by K-means with dynamic time warping. Red boxes show the significant structural changing windows (10-nt) between cluster 1 and cluster 2 pseudobulk reactivities. The shadow shows the structurally heterogeneous regions, in which 3 shadow regions are zoomed-in at bottom. Data are presented as mean values +/- SEM. **d**, Secondary structure model of 400 bases of RPL41 using the program RNAStructure and incorporating NAI-N3 pseudobulk reactivity in hESC (left) and NEU (right) as soft constraints. The bases that we strengthen are labeled as ‘lock’ in red (left) and ‘unlock’ in blue (right). **e**, Barplot showing luciferase units for WT and mutant RPL41 RNA structure in HEK293T cells after we locked (left) or unlocked (right) the structure. The center represents the mean, and the error bar shows the standard deviation. The *p*-values were calculated by one-tail Student’s T-Test. N = 8 biological replicates. Source data

As the same gene can exist in different conformations in different cells, we observed that the relative proportions of the structure populations in single cells can shift during differentiation and new structure conformations can emerge, resulting in observable aggregate structure changes during neuronal differentiation (Fig. 6b). To determine the functions of these structural shifts during neuronal differentiation, we overlapped transcripts with changes in structural heterogeneity and translation efficiency to identify 34 well-correlated genes (*R* (Spearman correlation) ≥0.9; Supplementary Table 4). One such gene encodes ribosomal protein L41 (RPL41), which shows an increased RNA structure heterogeneity and translation efficiency during neuronal differentiation (Fig. 6c and Extended Data Fig. 10a,b). Unsupervised clustering of RPL41 based on its structure reactivity identified two different structure populations during neuronal differentiation (Fig. 6d and Extended Data Fig. 10c,d) and showed that bases 160 and 310 exhibited structure differences in single cells (Fig. 6c). While the majority of hESCs contain high reactivity around RPL41 base 160, differentiated single cells tend to show lower reactivity for the same region (Fig. 6c,e). Conversely, a higher proportion of the differentiated single cells showed higher reactivity around the region in RPL41 base 310 than single cells from hESC (Fig. 6c,f). To determine whether base pairing around 160 indeed impacts RPL41 translation, we cloned the 5′ end of RPL41 in front of the luciferase reporter gene and performed mutagenesis experiments to either ‘lock’ or ‘disrupt’ the paired structure around base 160 (Extended Data Fig. 10d). Our luciferase experiments showed that ‘locking’ the structure at base 160 resulted in a slight increase in luciferase activity, while ‘unlocking’ it decreased luciferase activity, indicating that RNA structure can contribute to changes in RPL41 translation efficiency during neuronal differentiation (Extended Data Fig. 10e). These data suggest that RNA structure could serve as biomarkers in single cells to inform gene regulation.

## Discussion

Current methods for high-throughput RNA structure studies require millions of cells as starting material and cannot be used to study RNA structures in a very small number of cells. Here, we introduced sc-SPORT, a high-throughput approach to studying RNA structures in single cells. To do this, we optimized the protocol to identify conditions that increased mutation rates and efficiencies of library preparation. Importantly, fragmenting the RNAs in dNTPs helped us to concentrate RNA sizes to around 1,000 bases to enable efficient second-strand synthesis. Additionally, we developed a computational pipeline to analyze heterogeneous RNA structures, allowing us to identify them transcriptome wide.

We showed that RNA structure provides an additional layer of information in defining cellular identities and identified structurally variable regions in the transcriptome during different developmental states. We observed that RNA structures in hESC single cells are structurally more similar than RNA structures in differentiated cells. Structurally different regions are enriched in 3′ UTRs and associated with regulatory factors such as RBPs. Interestingly, we also observed a bimodal distribution in read density at the 3′ end of the transcripts in our single-cell analysis. We suspect that this bimodal distribution could be due to alternative 3′ UTR usage, which is a common form of gene regulation.

Importantly, we observed that RNA structural heterogeneity information can be used to inform RBP binding and gene regulation. We also observed that a heterogeneous region in the 3′ end of 18S rRNA is associated with translation. As this heterogeneous region is associated with the mRNA tunnel and can base pair with the mRNA during translation, we hypothesize that this variation reflects the translation status of the cell. As 18S rRNA structure can vary in individual cells due to biological states, we further hypothesize that this contributes to its slightly lower structural AUC–ROC of 0.6–0.7 in single cells, as compared with pseudobulk. As each cell can have its own unique structure and expression signature based on the cellular state that it is in, the combination of these two data types enriches our information on cellular identities.

One of the limitations of our current approach is that we capture only a few hundred cells (>300) in one experiment. Due to the need for manganese in our RT reaction, our protocol is incompatible with that used in 10X Genomics, making it challenging to directly apply 10X Genomics to scale up our cell numbers. However, we believe that coupling RNA structure probing with modifications to the 10X Genomics single-cell sequencing protocols or droplet sequencing will help to overcome this limitation in the long run.

Additionally, we did not incorporate UMI in our current protocol to remove PCR duplicates, as UMIs are added to the end of the transcript during RT and there is a fragmentation step before amplification. As such, only the reads at the end of the transcript will contain the UMI barcode. We confirmed that RNA structure reactivity is highly correlated with and without PCR duplicate removal, indicating that our data are robust to duplicate removal (Extended Data Fig. 2e–g). Further improvements in the protocol by using long-read sequencing can mitigate this shortcoming in improved versions of this method.

Overall, sc-SPORT transforms our understanding of RNA structure by revealing structure dynamics and regulation in single cells and in small rare cellular populations. Similar to single-cell RNA expression information, future applications of single-cell structure data can potentially enable us to cluster cells and predict developmental trajectories to better understand and refine structure–phenotype relationships in diverse biological systems.

## Methods

### Cell culture

H9 cells were cultured in mTeSR1 complete medium. To differentiate H9 cells into neurons^12^, we passaged H9 cells using dispase at a dilution of 1:10 and induced H9 cells using neural induction media (3 μM CHIR99021, 2 μM SB431542, 0.1 μM compound E, 20 ng ml^−1^ epidermal growth factor (EGF), 20 ng ml^−1^ basic fibroblast growth factor (bFGF)) 1–2 days later. H9 cells were changed with fresh medium every 1–2 days. The cells were then split 1:3 using accutase and reseeded on Matrigel-coated plates 7 days later before culturing in a neural cell culture medium. These derived cells are NPCs, which were used for further neuron differentiation. Neuron progenitor cells were cultured in neuron differentiation medium (10 ng μl^−1^ brain-derived neurotrophic factor, 10 ng μl^−1^, glial cell line-derived neurotrophic factor, 300 ng ml^−1^ cAMP and 0.2 mM vitamin C) for 1 day to obtain iNeus and 7 days to obtain NEUs.

### Sc-SPORT experimental workflow

We dissociated cells into a single-cell suspension and incubated them with a final concentration of 25 mM NAI-N3 for 10 min at 37 °C with constant rotation. We then removed the excess NAI-N3 from the cells by centrifugation for 5 min at 300*g*. We resuspended the cells in cold phosphate-buffered saline and diluted the cells to around 100 μl^−1^ before picking single cells by mouth pipetting under a microscope (<0.5 μl). We then transferred the cells into a prepared eight-strip tube, with each cell in one tube. The eight-strip tube contains 3.5 μl of fragmentation and annealing buffer (0.2 U μl^−1^ SUPERase In RNase Inhibitor, 1 μM oligo dT (5′-AAG CAG TGG TAT CAA CGC AGA GTA CT_30_VN-3′) and 1 mM dNTP). We placed the eight-strip tube in a thermocycler and ran the fragmentation and primer annealing program (95 °C for 10 min, 4 °C for 10 min, 72 °C for 3 min, 4 °C for 10 min and 4 °C hold). After primer annealing, we added 6 μl of RT reaction mix into each tube and mixed it gently. The composition of the RT reaction mix is 1× first-strand buffer ((50 mM Tris pH 8.0, 75 mM KCl), 1 μM TSO (5′-AAG CAG TGG TAT CAA CGC AGA GTA CAT rGrGrG-3′), 1 U μl^−1^ SUPERase In RNase Inhibitor, 5 mM DTT, 6 mM MnCl_2_, 10 U μl^−1^ SSII and 1 M betaine). We then ran the RT program using the following conditions: 25 °C for 5 min, 42 °C for 8 h, 70 °C for 10 min and 4 °C hold.

After RT, we added the PCR reaction mix (working concentration of 0.1 μM ISPCR primer (5′-AAG CAG TGG TAT CAA CGC AGA GT-3′) and 1× HIFI KAPA master mix) to each tube and ran the following PCR program: 98 °C for 3 min (98 °C for 20 s, 67 °C for 15 s and 72 °C for 3 min) and 72 °C 5 min), for 24–26 cycles for NAI-N3-treated samples and 22 cycles for dimethyl sulfoxide (DMSO)-treated samples. The amplified PCR products were analyzed using an Agilent bioanalyzer 2100.

After PCR, PCR products were purified using Ampure XP beads twice, before being used for library preparation using the Illumina Nextra XT kit. Briefly, we diluted each sample to 0.6–0.8 ng μl^−1^ and transferred 1 μl of each sample into a 96-well PCR tube. We then added 2 μl of the Tagmentation DNA buffer and 1 μl of amplicon tagmentation mix to the sample in the 96-well PCR tube and incubated the reaction at 55 °C for 5 min. We immediately added 1 μl NT buffer from the Illumina Nextra XT kit to the mixture, mixed it up and down, and incubated the mixture at room temperature for 5 min to stop the tagmentation reaction. We then added 1 μl of i5 and 1 μl of i7 barcoded primers before adding 3 μl of NPM from the Illumina Nextra XT kit to each tube. We then ran the following PCR program: 72 °C for 3 min, 95 °C for 30 s (95 °C for 10 s, 55 °C for 30 s and 72 °C for 30 s), 72 °C for 5 min and hold at 10 °C). The prepared libraries were sequenced using Illumina Hi-Seq 4000 (sequencing type 2× 150).

### Generating RNA structure data from 10 and 100 cells

Instead of picking a single cell by mouth pipetting, we diluted the cells into 20 cells μl^−1^ for ten-cell library preparation (200 cells μl^−1^ for 100 cells) and transferred 0.5 μl of the mixture into prepared fragmentation and primer annealing buffer for downstream library construction.

### Preparation of in vitro and in vivo structural benchmarks

For structure mapping of the Tetrahymena ribozyme in vitro, we PCR amplified the Tetrahymena ribozyme DNA that contains the T7 promoter (Supplementary Table 6) upstream of the ribozyme. The DNA template was then in vitro transcribed using the NEB HiScribe Kit following the manufacturer’s instructions to generate the Tetrahymena ribozyme RNA.

To fold the Tetrahymena ribozyme in vitro, we heated the 1 μg RNA in 9 μl water at 90 °C s^−1^ for 2 min and chilled it on ice immediately for 2 min. We then added 1 ul 10× RNA structure folding buffer (500 mM Tris pH 7.4, 100 mM MgCl_2_ and 1.5 M NaCl) to the RNA on ice, slowly increased the temperature to 37 °C at 0.1 °C s^−1^ and incubated the RNA at 37 °C for 20 min. To perform structure probing of the RNA in vitro, we added NAI-N3 (homemade), 2-methylnicotinic acid imidazolide (NAI) (913839, Sigma) or dimethyl sulfate (DMS) (D186309, Sigma) individually (Fig. 1b,c) to the folded RNA and incubated the reaction at 37 °C for 10 min. We also performed a separate reaction using DMSO as a negative control for NAI-N3, NAI or DMS treatment. The structure probed RNA was purified using phenol:chloroform:isoamyl alcohol (25:24:1) and reverse transcribed before either running out on a sequencing gel or made into a sequencing library for high-throughput sequencing.

For structure probing of the Tetrahymena ribozyme and two other ribosNitches and their MTs (Supplementary Table 6) inside cells (Fig. 2f–i and Extended Data Fig. 5a–e). We pooled the three in vitro-folded WT RNAs as sample pool 1 and their MT RNAs as pool 2 and then transfected each RNA pool into HEK293T cells using Lipofectamine MessengerMAX transfection reagent (Thermo Fisher, LMRNA015). We then dissociated the cells 6 h after transfection and washed them three times using PBS to remove the excess RNA that did not enter the cells. We treated the cells with either 25 mM NAI-N3 or DMSO for 10 min before performing single-cell RNA structure probing by picking individual cells into each well of a 96-well plate using mouth pipetting.

### Overexpression of PUM2 in hESCs

We generated a Tet-On PUM2 expression system in H9 hESCs^12^ (Fig. 4e and Extended Data Fig. 7f). Lentiviral vectors (tetO–PUM2, pMDLg/pRRE, pRSV–REV and pMG2.G) were cotransfected into HEK–293T cells. We changed the medium after 24 h posttransfection and kept culturing the cells for another 24 h before collecting the virus. We then infected H9 hESCs after concentrating the virus particles using centrifugal filters (Ultracel-100K, UFC910096).

### Sc-SPORT data processing and analysis

We trimmed reads to remove adapter contamination and poor-quality reads using the software cutadapt (version 1.8.1) (ref. ^37^). The trimmed reads were then mapped to the human transcriptome using bowtie2 (version 2.2.6) (ref. ^38^) according to the longest coding region transcriptome annotation based on the human genome (GRCh38 and Ensembl 104 and Gencode version 38). The mutations were identified using bam-readcount (version: 0.8.0) and custom scripts together. The reactivities were then calculated by subtracting mutational rates in DMSO-treated samples from mutational rates in NAI-N3 structure probed samples.

The expression levels of each transcript were quantified using Salmon (v1.1.0) (ref. ^39^) with the annotation from ENSEMBL (GRCh38 release 98). Following the quantification, expression levels were normalized using the method described by Hafemeister and Satija^40^. On the basis of the normalized expression levels, cell cycle assignments were performed using Seurat (version: v3.6.3) (ref. ^41^). The alternative splicing per transcript was quantified by proportion spliced-in using SUPPA2 (version: v2.3) (ref. ^42^) based on the transcripts per million values estimated by Salmon. As a quality control, we removed cells with (1) more than 5% of reads mapped to mitochondrial genes and (2) fewer than 5,000 detected genes. The rest of the cells were used for downstream analysis.

### Calculating window-level structural heterogeneity along a transcript

We designed a computational pipeline to quantitate the amount of structural heterogeneity in the transcripts as follows: (1) we split the transcriptome into discrete windows of 10 nt in length; (2) we only considered a window as detected when it has a total coverage of more than 600 reads. This threshold was set by comparing the correlation between biological replicates at different sequencing depths (Extended Data Fig. 3b); and (3) we filtered away windows that are detected in less than 50% of cells. We then calculated an NAI-N3 modification rate by summing up the total read coverage and the total read number with mutations across a window of 10 nts. To calculate window-level structural heterogeneity, we assumed that the MT reads and the read coverage of each 10 nt window follow a simple linear model. The *R*^2^ of the linear model could be used as a measure of the deviation of the reactivities in each cell to their average reactivity. For each window:

$${\rm{mutant}}\left({{c}},{{s}}\right)=\mathrm{mod}\left({{s}}\right)\times {\rm{depth}}\left({{c}},{{s}}\right)+\varepsilon$$

where MT(*c*, *s*) is the number of MT reads in a cell *c* at the window *s*, the mod(*s*) is the expected modification rate by NAI-N3 at the window *s* and the depth(*c*, *s*) is the total read coverage in cell *c* at the window *s*.

We calculated *R*^2^ of the simple linear model using the python package scipy.stats.lineargress. As a perfectly homogeneous window will have the same modification rate by NAI-N3 in all cells, the proportion of variance explained by the *R*^2^ to the linear model should be close to 1. On the other hand, a heterogeneous window will have different modification rates; thus the *R*^2^ is close to 0. To account for the missing data in single-cell sequencing, we used the adjusted *R*^2^ (adj *R*^2^) as the measurement of structural heterogeneity at the window level (Figs. 3–6 and Extended Data Figs. 6e–l,7g,8,9).

### Calculating gene-level structural heterogeneity in the transcriptome

We determined the extent of structural heterogeneity for each transcript by comparing the variation of structural reactivities in each cell to the pseudobulk reactivity of all cells. We applied a quantile normalization to the raw reactivity to minimize putative differences in modification efficiencies in single cells. We then computed the cosine distances (*D*_*i*_) for each transcript in each cell (*R*_*i*_) against its pseudobulk reactivity (*R*_pseudo_) (Fig. 3a,d,f and Extended Data Fig. 7a). The pairwise cosine distance was calculated using sklearn.metrics.pairwise.cosine_distances.

$${D}_{{C}_{i}}={D}_{C}\left({R}_{i},{R}_{{\mathrm {pseudo}}}\right)=\frac{\left({R}_{i}\cdot {R}_{{\mathrm {pseudo}}}\right)}{\left(\left|\left|{R}_{i}\right|\right|\times \left|\left|{R}_{{\mathrm {pseudo}}}\right|\right|\right)}$$

The dispersion of the pairwise cosine distances is used as the measurement for transcript-level structural heterogeneity. The *n* represents the number of cells in the population.

$${\rm{Heterogeneity}}=\sqrt{\frac{\mathop{\sum }\nolimits_{i=1}^{n}{D}_{{C}_{i}}^{2}}{n}}$$

### Calculating AUC–ROC for positive controls

To calculate AUC–ROC using 18S rRNA and the Tetrahymena ribozyme as positive controls, we classified single-stranded nucleotides in their secondary structures as ‘True’ for truly modified and double-stranded ones as ‘False’ for falsely modified bases. We plotted the AUC–ROC curve and calculated the AUC–ROC score using reactivities of each nucleotide against the secondary structures using the *roc_curve* and *roc_auc_score* functions in scikit-learn (v1.0.2) package. For 18S rRNA, we used the protein data bank (PDB) structure (id: 6ek0) to calculate the solvent accessibility for 2′-OH of each nucleotide^43^. The bases with solvent accessibility ≥3 were then used to calculate the accuracy (Fig. 3b,c).

We also evaluated the distribution of reactivities at bases located base paired between adjacent base pairs, paired in terminal base pair and in unpaired regions of the Tetrahymena group I intron (Extended Data Fig. 1a) and 18S rRNA secondary structures (Extended Data Fig. 4b)^44^. The reactivities in the paired regions are significantly lower than the reactivities in the unpaired regions, indicating that our single-cell structure probing method is accurate.

### Enrichment analysis

The binding sites of 183 different RBPs determined by eCLIP^24^ were downloaded from ENCODE. The binding sites of microRNA were downloaded from TargetScan version 7.2 with default predictions^45^.

To calculate enrichment, we overlapped our windows of interest and background with the RBP binding windows and calculated the significance of the overlap using a hypergeometric test. The resulting *P* values were adjusted using the Bonferroni method. We identified a regulator as enriched when they have an adjusted *P* value ≤0.05 (Fig. 4a,b and Extended Data Figs. 7b and 9b).

### MOFA

MOFA^46^ was used to jointly infer the variation from multiple data types. We used multiple combinations of regulatory layers to train different models in MOFA: expression only (EXP), expression and splicing (EXP + AS), expression and structural heterogeneity (EXP + STR), and finally expression, splicing and structural heterogeneity (EXP + AS + STR) (Fig. 6a). The expression layer includes the top 1,000 most variably expressed genes across all cells based on the normalized expression levels. The splicing layer includes the top 1,000 most variably spliced transcripts across all cells based on proportion spliced-in values. The structural heterogeneity layer contains 238 genes detected in all four neuronal differentiation stages. Training of the model was carried out using the default parameters. The latent factors inferred by MOFA were used to cluster the cells using *k*-means clustering with four predefined clusters. The clustering results were then compared to how cells are distributed in the four differentiation stages. An adjusted rand index (ARI) value was calculated to evaluate the clustering results against biological differentiation stages.

### Structural modeling

Secondary structures were modeled by incorporating structural reactivities in the program RNAstructure (v6.3) (ref. ^47^). Briefly, we modeled the structure by incorporating SHAPE reactivity with sequence information. We used the function Rsample to calculate the partition function and generated a Boltzmann ensemble of 1,000 structures. We then used RsampleCluster.R applied to calculate the optimal number of clusters and their centroid structures (Extended Data Fig. 10d).

### Quantification and statistical analysis

All statistical analyses were performed in R (version 3.6.3) or Python (3.10.0) unless otherwise stated in the methods. Students’ *t*-tests were performed using the t.test() function in R. The hypergeometric tests were performed using the phyper() function in R. The hypergeometric tests were performed using the phyper() function in R and scipy.stats.hypergeom() function in Python. The nonparametric statistic test was performed using scipy.stats.mannwhitneyu in Python. The error bars in the line plots of reactivity are standard errors of each nucleotide among cells.

### Reporting summary

Further information on research design is available in the Nature Portfolio Reporting Summary linked to this article.

## Online content

Any methods, additional references, Nature Portfolio reporting summaries, source data, extended data, supplementary information, acknowledgements, peer review information; details of author contributions and competing interests; and statements of data and code availability are available at 10.1038/s41592-023-02128-y.

### Supplementary information

Supplementary Information Supplementary Tables 1–6.

Reporting Summary

Descriptions of the supplementary tables.

### Source data

Source Data Fig. 1 Statistical source data.

Source Data Extended Data Fig. 1/Table 1 Unprocessed SAFA gels.

Source Data Extended Data Fig. 1/Table 1 Statistical source data.

Source Data Extended Data Fig. 4/Table 4 Unprocessed SAFA gels.

Source Data Extended Data Fig. 5/Table 5 Unprocessed SAFA gels.

Source Data Extended Data Fig. 10/Table 10 Statistical source data.

## Data Availability

The sequencing data were uploaded to the Sequence Read Archive at the National Center for Biotechnology Information. The accession numbers are PRJNA946372, PRJNA946273 and PRJNA946308. The translation efficiency and RNA decay data in human neural differentiation come from the previous paper. Transcript efficiency and RNA decay data used in this paper were from Wang, J. et al.^12^. Source data are provided with this paper.

## Code availability

Analysis code is available at https://github.com/noahpieta/scSports. See the details of data analysis in Methods.
