# Supplementary material for: RNA structure profiling at single-cell resolution reveals new determinants of cell identity
Source: Nat Methods. 2024 Jan 4;21(3):411–22. doi: 10.1038/s41592-023-02128-y (PMC10927541; doi:10.1038/s41592-023-02128-y)
Supplement: Supplementary file 1 — Supplementary Tables 1–6. [file 41592_2023_2128_MOESM1_ESM.pdf]

nature methods

Article https://doi.org/10.1038/s41592-023-02128-y

RNA structure profiling at single-cell resolution reveals new determinants of cell identity

In the format provided by the authors and unedited

The Description of Tables S1–S6

Supplementary table 1

The sequencing mappability of each sample (single-cell or mini-bulk) at different time points of batch 1 and batch 2.

Supplementary table 2

The RNA structure heterogeneity of each gene is calculated based on the cosine distance (gene level).

Supplementary table 3

Unsupervised clustering of RNA windows based on their structural heterogeneity (window level R^2).

Supplementary table 4

The well-correlated genes between RNA structure changes and their translation changes during the neurogenesis process.

Supplementary table 5

The genes that have both RNA structure changes and RNA half-life changes in hESCs and NPCs

Supplementary table 6

The sequence of benchmark genes (wildtype and their mutant) which were transfected in HEK293T cells.
