# Supplementary figures and images for: RNA structure profiling at single-cell resolution reveals new determinants of cell identity
Source: Nat Methods. 2024 Jan 4;21(3):411–22. doi: 10.1038/s41592-023-02128-y (PMC10927541; doi:10.1038/s41592-023-02128-y)

# Source data of Extended data Figure 1

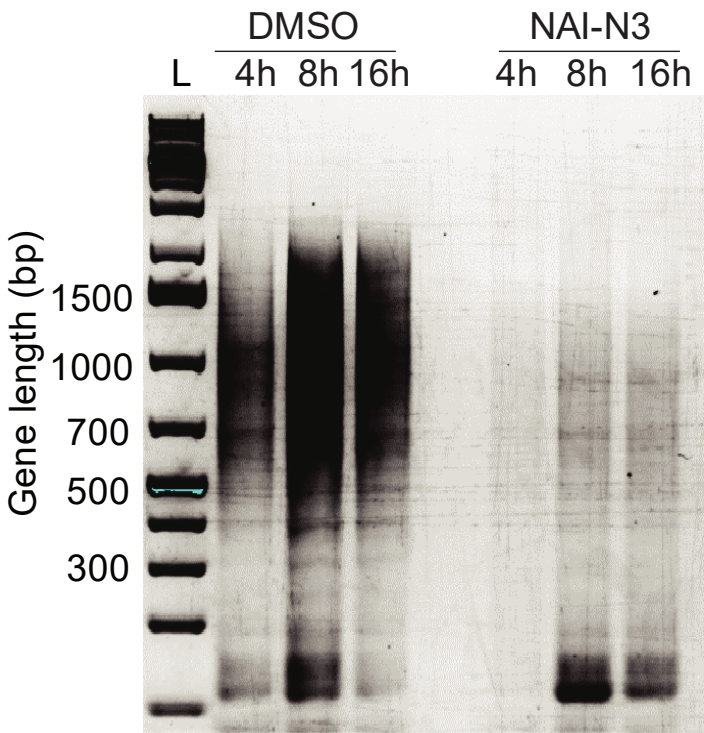

Supplement: Supplementary file 5 — Unprocessed SAFA gels. [file 41592_2023_2128_MOESM5_ESM.pdf]

Source data of Extended data Figure 4

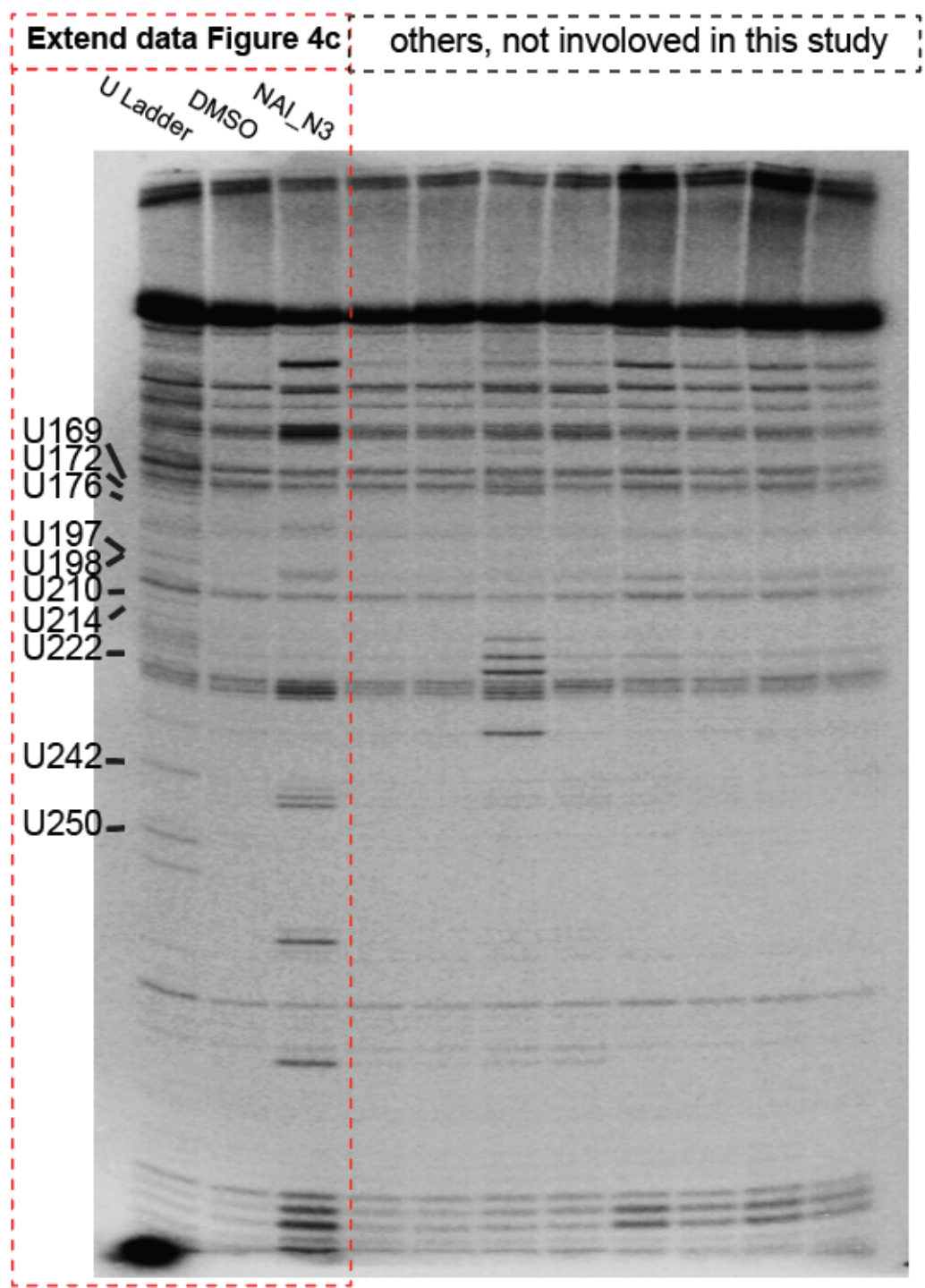

Supplement: Supplementary file 7 — Unprocessed SAFA gels. [file 41592_2023_2128_MOESM7_ESM.pdf]

source data of Extended data Figure 5

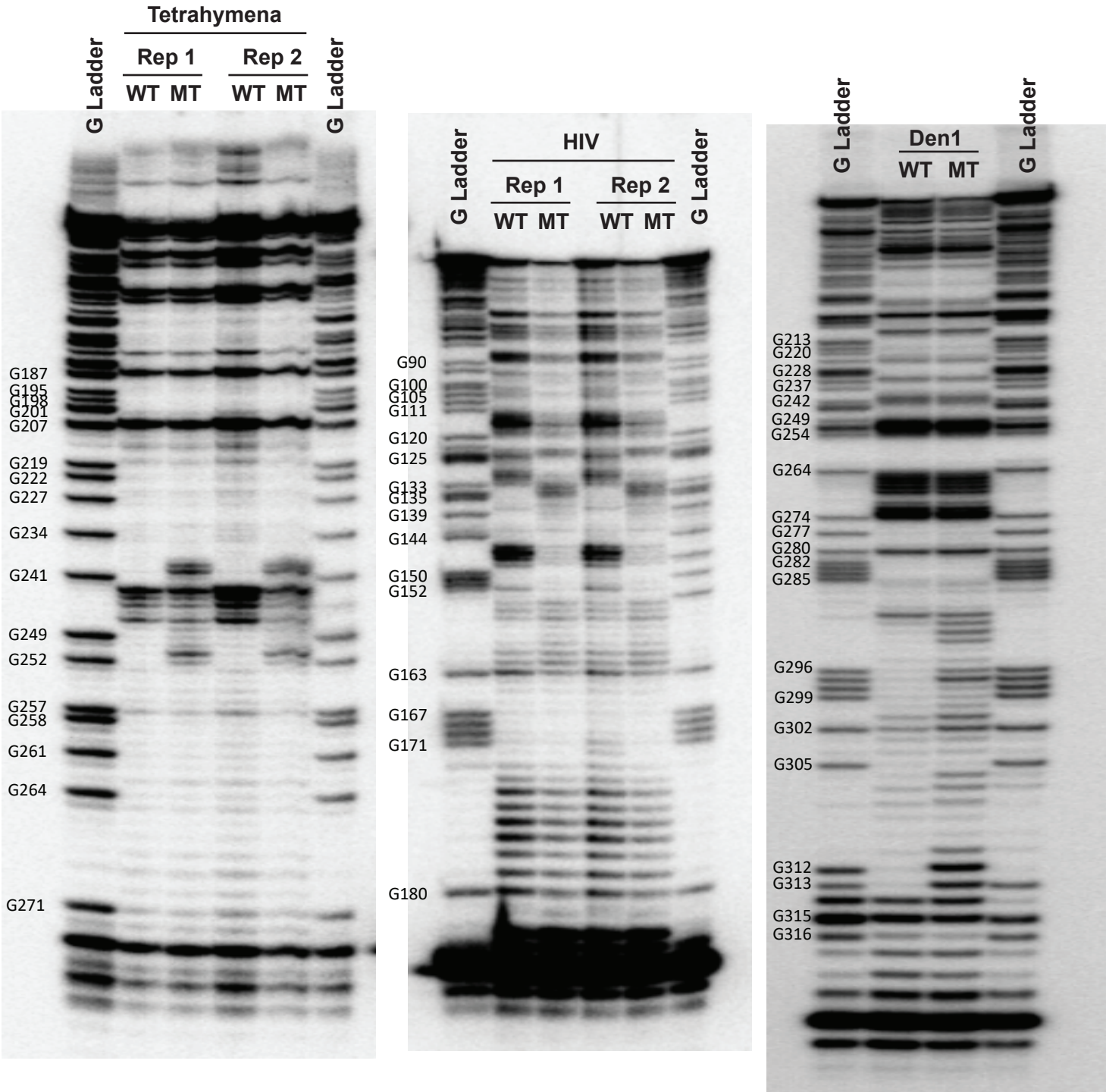

Supplement: Supplementary file 8 — Unprocessed SAFA gels. [file 41592_2023_2128_MOESM8_ESM.pdf]
